# Conjugative Stabilization versus Anchimeric Assistance in Carbocations

**DOI:** 10.3390/molecules28010038

**Published:** 2022-12-21

**Authors:** Bagrat A. Shainyan

**Affiliations:** A.E. Favorsky Irkutsk Institute of Chemistry, Siberian Branch, Russian Academy of Sciences, 1 Favorsky Str., 664033 Irkutsk, Russia; bagrat@irioch.irk.ru

**Keywords:** carbocations, conjugative stabilization, anchimeric assistance, structure, rearrangements

## Abstract

In this study, an old concept of anchimeric assistance is viewed from a different angle. Primary cations with two different heteroatomic substituents in the α-position to the cationic carbon atom CHXY–CH_2_^+^ (X, Y = Me_2_N, MeO, Me_3_Si, Me_2_P, MeS, MeS, Br) can be stabilized by the migration of either the X or Y group to the cation center. In each case, the migration can be either complete, resulting in the transfer of the migrating group to the adjacent carbon atom and the formation of a secondary carbocation stabilized by the remaining heteroatom, or incomplete, leading to an anchimerically assisted iranium ion. For all combinations of the above groups, these transformations have been studied by theoretical analysis at the MP2/aug-cc-pVTZ level and were shown to occur depending on the ability of anchimeric assistance by X and Y, as well as the conformation of the starting primary carbocation. In the conformers of α-amino cations with the *p*-orbital, C–N bond and the nitrogen lone pair in one plane, the Me_2_N group migrates to the cationic center to give aziranium ions. Otherwise, the second heteroatom is shifted to give iminium ions, without or with very slight anchimeric assistance. In the α-methoxy cations, the MeO group can be shifted to the cationic center to give the O-anchimerically assisted ions as local minima, the global minima being the ions anchimerically assisted by another heteroatom. The electropositive silicon tends to migrate towards the cationic center, but with the formation of a π-complex of the Me_3_Si cation with the C=C bond rather than a Si-anchimerically assisted cation. The phosphorus atom can either fully migrate to the cationic center (X = P, Y = S, Se) or form anchimerically stabilized phosphiranium ions (X = P, Y = O, Si, Br). The order of the anchimeric assistance for the heaviest atoms decreases in the order Se >> S > Br.

## 1. Introduction

The concept of anchimeric assistance (or acceleration of a reaction due to neighboring group participation) is over 60 years old [1,2]. It has also been termed *synartetic acceleration* [3,4,5] and, in the chemistry of carbocations, *non-vertical stabilization* [6,7]. Over the past decades, the concept has been broadened and enriched by new data. In addition to the neighboring aryl or other carbon-centered groups [1,2,3,4,5,6,8,9], the anchimeric assistance by many other juxtaposed atoms and groups has been studied. For illustration, the anchimeric assistance of nitrogen [10,11,12,13], oxygen [14,15], halogens [16,17], and even hydrogen atoms [18] can be mentioned. Well-documented are the abilities of various metals —chromium [19], platinum [20], palladium [21], vanadium [22], and many others—to facilitate different reactions by anchimeric assistance. Well-known is anchimeric assistance in the hydrolysis of yperite and nitrogen yperite [23,24], in which the generated S or N three-membered rings strongly accelerate the reaction, as well as in the reactions of triphenylphosphine with α,β-unsaturated carboxylic acids [24] and bifunctional tertiary phosphines with activated alkenes [25]. The mechanism of anchimeric assistance was also analyzed theoretically at the DFT level [12,26,27,28] and proved experimentally [9,29,30]. Of most relevance to the present work are the studies on anchimeric assistance by silicon- [31,32], phosphorus- [33], sulfur-, and selenium-containing groups [7,13,34,35], which shows a pronounced stabilizing effect on the carbocationic intermediates.

### Computational Details

All calculations were performed with full geometry optimization using the MP2/aug-cc-pVTZ method as implemented in Gaussian 09 [36]. Conditionally, cations with incomplete transfer of group X or Y were considered as “iranium” when the C–C–X(Y) angle was less than 100° and as “open” when it was more than 100° and close to the tetrahedral angle of 109.5°. Of course, any “demarcation” between the “iranium” and “open” forms is conditional, but the deviation of this angle from the tetrahedral value of ten to fifty degrees can only be due to attractive interaction between the X or Y group and the adjacent carbocationic center, while a deviation of only several degrees is insignificant and does not imply any notable interaction. Apart from the “iranium” or “open” structure obtained during geometry optimization with the shift of group X, the isomeric cations corresponding to the complete or incomplete transfer of group Y were calculated as local or global minima on the PES. Since the course of optimization depends on the relative orientation of the empty *p*-orbital on the carbocation center and the C–X bond, as well as the lone pair on heteroatom X, different starting geometries of the primary cations were examined. The total energies of the stationary points optimized as global or local minima were compared. No frequency calculations were performed because of the extremely high computational costs at such a high level of theory.

## 2. Results and Discussion

**Figure 1:**
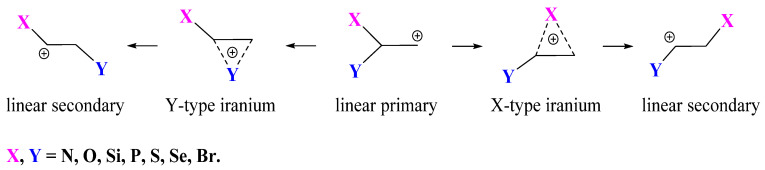
Stabilization of the primary cation with respect to the iranium cation by anchimeric assistance or to the secondary cation by 1,2-shift of the substituent.

In the present paper, the problem of anchimeric assistance is studied from a new perspective, namely, by investigation of the relative ability of different groups attached to the same carbon to stabilize the adjacent primary carbocationic center either by anchimeric assistance via the formation of the X- or Y-type iranium cation or via full migration to this carbon atom, forming linear secondary cations, as shown in Figure 1.

For this, high-level theoretical calculations were performed for all 21 combinations of substituents X and Y, including both global and local minima corresponding to the iranium and linear cations presented in Figure 1. In order to avoid possible isomerization via hydrogen migration from the XH or YH group, all heteroatoms were protected by methyl groups (OMe, SMe, SeMe, NMe_2_, PMe_2_, and SiMe_3_), which allowed for the analysis of “pure” anchimeric assistance or migration of X or Y. Among the issues addressed in this study are the dependence of the type of the migrating group and the degree of its shift towards the carbocationic center on the nature of the migrating and remaining groups X and Y, the reasons for the formation of local (kinetically controlled) and global (thermodynamically controlled) products on the potential energy surface (PES), and the relative effectiveness of anchimeric assistance versus stabilization by direct conjugation with the lone pair of a heteroatom.

The main factors affecting the degree of anchimeric assistance are the covalent radius of the interacting group and its polarizability. The larger the covalent radius, the longer the distance at which the migrating group interacts effectively with the carbocationic center. The larger the polarizability, the easier it is to induce a dipole, and hence the larger the binding energy. The ability of a specific group to stabilize the existing or incipient charge on the adjacent carbon atom is usually estimated by comparing the rates of the reactions of the parent and substituted compounds. By contrast, in the present work, the relative abilities of different groups to stabilize the positive charge are estimated from the relative stabilities of the structures formed by full or incomplete transfer of one of two substituents in the same molecule. This raises four main questions that are answered in this paper: (i) that of which group migrates spontaneously during geometry optimization; (ii) that of which factors are responsible for the migration of a particular group; (iii) that of which stabilization is preferable: anchimeric assistance with the partially shifted group X or direct conjugation with the lone pair on the non-migrating group Y; and (iv) that of the structure of the formed cations (covalently bound iranium ions, anchimerically assisted ions, or π-complexes with practically intact double bonds). The primary cations in Figure 1 are inherently unstable and in all cases are stabilized by group X or Y to either iranium or secondary cations. Below, the transformations of primary cations with all possible combinations of groups X and Y will be considered. 

**Scheme 1:**
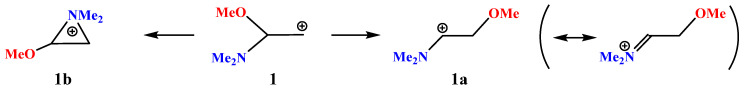
Iminium **1a** versus aziranium **1b** cation formation.

**X = NMe_2_, Y = OMe (1).** The neighboring amino group participation may result in the formation of the rearranged products via the formation of the anchimerically assisted aziridinium ion. For example, as applied to our case, the amino group wins the competition with iodine, as proved by the intramolecular ring opening of the preliminarily formed halogen-assisted cation [37]. The result of geometry optimization of cation **1** depends on the relative orientations of the Me–N–Me and N–C–CH_2_ planes. If they are perpendicular, that is, if the *p*-orbital and the nitrogen lone pair are eclipsed (lie in the same plane), the aziranium cation **1b** is formed; for all other conformations, full migration of the methoxy group is observed, resulting in the iminium cation **1a**, as shown in Scheme 1. 

**Figure 2:**
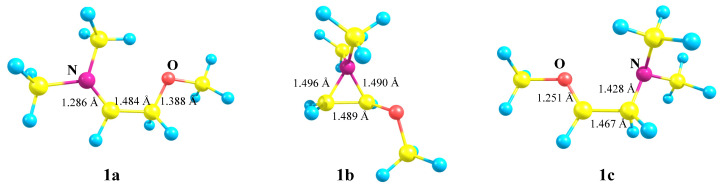
Optimized geometries of the iminium **1a**, aziranium **1b**, and oxenium **1c** cations.

No isomeric oxiranium cation analogous to **1b** could be located. However, linear oxenium cation **1c** was found to be at a local minimum on the PES (Figure 2). The relative energies increased in the order **1a** (0) < **1b** (10.0) < **1c** (28.5 kcal/mol) (see Supplementary Materials).

The geometries of the located minima are given in Figure 2. Strong N and O stabilization of the secondary cations **1a** and **1c** is evidenced by ~0.14 Å shortening of the N–C^+^ and O–C^+^ bonds. In both **1a** and **1c**, the C–C bond length is close to that of the ordinary bond, suggesting their existence as iminium (**1a**) or oxenium (**1c**) cations. The 0.017 Å longer C–C bond in **1a** relative to that in **1c** is indicative of stronger N versus O stabilization. The lengths of the C–N bonds in **1a** and in the almost equilateral triangle of **1b** fall in the range of normal ordinary C–N bonds.

Therefore, the relative orientation of the C–N bond and the nitrogen lone pair with respect to the empty *p*-orbital on the carbocation center in **1** has a strong impact on the migration ability of the NMe_2_ group. 

The Mulliken charge distribution in the aziranium cation **1b** is strongly polarized: *q*_N_ = +0.621, *q*_CH2_ = −0.683, and *q*_CH_ = +0.101, suggesting the covalent nature of the N–CH bond and the electrovalent nature of the N–CH_2_ bond.

**Scheme 2:**
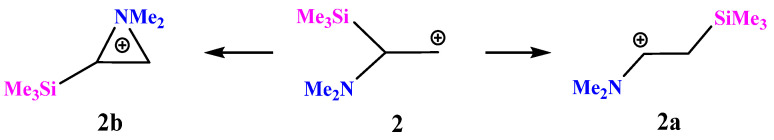
Iminium **2a** versus (trimethylsilyl)aziranium **2b** cation formation.

**X = NMe_2_, Y = SiMe_3_ (2).** For the same reasons, optimization of cation **2** gives rise to the iminium cation **2a** via the migration of the Me_3_Si group for all initial conformations except the one that has both the Me_2_N–C bond and the nitrogen lone pair in the same plane with the cationic *p*-orbital. In the latter case, aziranium cation **2b** was located on the PES as the local minimum lying 20.8 kcal/mol above the global minimum of **2a**, Scheme 2. 

The planar structure of the NC_3_ fragment and the tetrahedral C–C–Si angle of 109° proves the absence of interaction of the silicon atom with the cationic center in **2a**. The aziranium triangle in **2b** is close to equilateral. 

The aziranium cation **2b** is polarized more strongly than its methoxy-substituted analogue **1b** due to the electron donor effect of the trimethylsilyl group: *q*_N_ = +0.706, *q*_CH2_ = −0.822, and *q*_CH_ = −0.315. No siliranium cation was found on the PES.

**Scheme 3:**
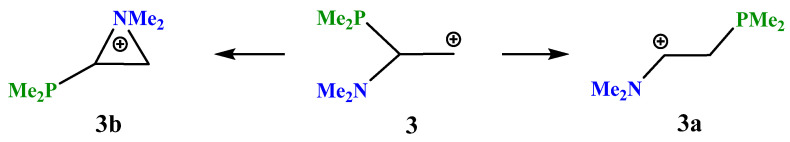
Iminium **3a** versus (dimethylphosphino)aziranium **3b** cation formation.

**X = NMe_2_, Y = PMe_2_ (3).** The transformations of cation **3**, which has two heteroatoms belonging to the same group of the periodic table (Scheme 3), are completely similar to those of cation **2**, except that the energy difference between the global minimum of the iminium cation **3a** and the local minimum of the aziranium cation **3b** is notably smaller: 16.1 kcal/mol. Apparently, this is due to the practically equal lengths of all three P–C bonds in **3b** (Δ*l*~0.01 Å), whereas in its analogue **2b** the Si–C bonds with the methyl groups are ~0.06 Å shorter than in the CNC ring carbon atom.

The planar structure of the NC_3_ fragment and the nearness to the tetrahedral C–C–P angle of 108° proves the iminium structure of cation **3a** and the absence of interaction of the phosphorus atom with the cationic center (*l*(P–C^+^) = 2.743 Å). 

The aziranium cation **3b** is polarized even more strongly than its trimethylsilyl-substituted analogue **2b**: *q*_N_ = +0.762, *q*_CH2_ = −0.858, and *q*_CH_ = 0.302. Note that, unlike **2b**, the CH carbon in **3b** is charged positively, apparently, due to the higher electronegativity of phosphorus compared to silicon. In line with this, the N–C bond between the oppositely charged atoms in **3b** (1.480 Å) is shorter than that between the likely charged ones (1.504 Å). No phosphiranium cation was found on the PES.

**Scheme 4:**
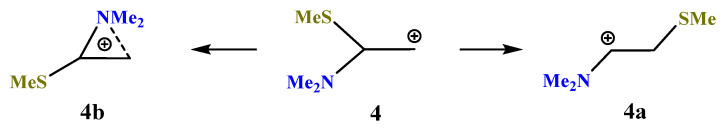
Iminium **4a** versus (methylthio)aziranium **4b** cation formation.

**X = NMe_2_, Y = SMe (4).** Moving further along the third row of the periodic table (Si → P → S) results in a further decrease in the energy gap between the global **4a** (linear iminium cation) and local **4b** (aziranium cation) minima on the PES (Scheme 4). As above, the primary cation **4** is optimized to the aziranium cation **4b** only for the starting conformation of **4** with the *p*-orbital, C–N bond and the lone pair on nitrogen lying in one plane. The value of Δ*E* = 12.1 kcal/mol is minimal in the series of all Me_2_N-containing carbocations (**1**)–(**6**).

**Figure 3:**
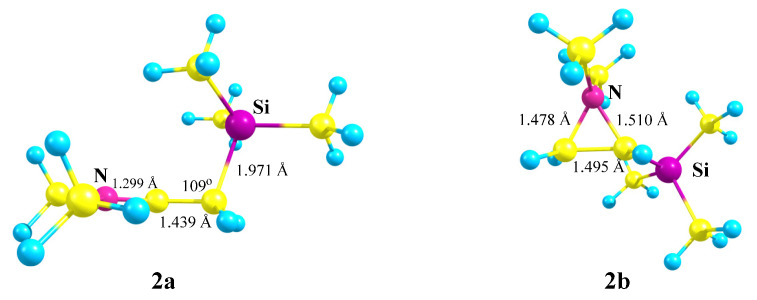
Optimized geometries of the iminium **2a** and aziranium **2b** cations.

**Figure 4:**
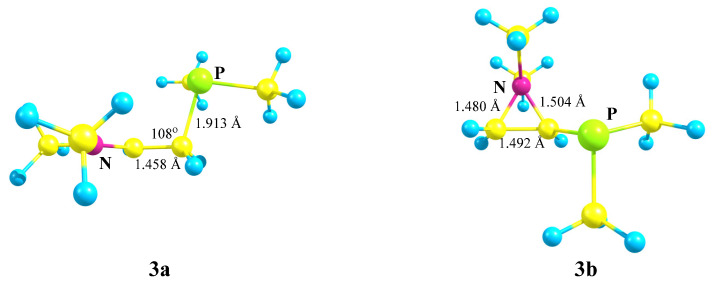
Optimized geometries of the iminium **3a** and aziranium **3b** cations.

**Figure 5:**
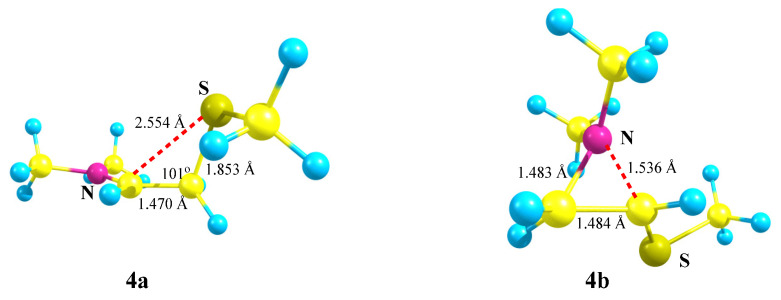
Iminium cation **4a**, with reduced CCS angle and C⋯S distance, and unsymmetrical aziranium cation **4b**.

Note the appearance of the signs of anchimeric assistance of the sulfur atom in **4a**, as evidenced by a smaller C–C–S angle of 101° in **4a** as compared to the tetrahedral angles of C–C–Si and C–C–P in **2a** and **3a** (Figure 3, Figure 4 and Figure 5). The non-covalent C–S distance in **4a** is 2.554 Å, which is larger than the covalent bond length but much less than the sum of the van der Waals radii (3.5 Å) [38]. Note, also, the less symmetrical structure of the CNC triangle in **4b**, in particular, the larger difference in the angles and, especially, the C–N distances in the ring (0.053 Å).

The Mulliken charge on nitrogen and the polarization of the aziranium cation **4b** (*q*_N_ = +0.687, *q*_CH2_ = −0.769, and *q*_CH_ = +0.121) are somewhat higher than those in its oxygen analogue **1b** but lower than those in **2b** and **3b**.

**Scheme 5:**
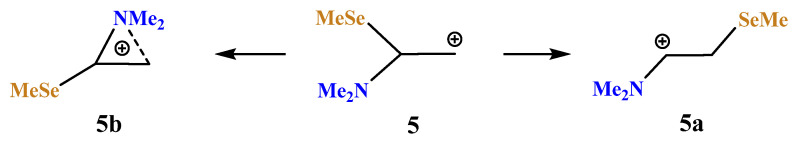
Iminium **5a** versus (methyselanyl)aziranium cation **5b** formation.

**Figure 6:**
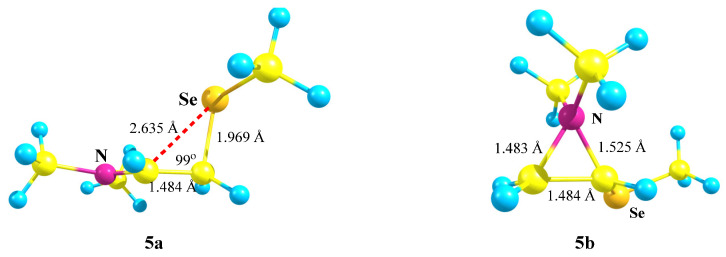
Immonium cation **5a**, with reduced CCSe angle and C⋯Se distance, and unsymmetrical aziranium cation **5b**.

**X = NMe_2_, Y = SeMe (5).** Introduction of a heavier chalcogen, selenium, in place of sulfur, has a small effect on the structure and energetics of the corresponding carbocations (Scheme 5, Figure 6). The comparison of the covalent and non-covalent distances and bond angles allows the conclusion that the anchimeric assistance in the aziranium cation **5b** must be somewhat stronger than in its analogue **4b**. However, the calculated energy difference of 15.0 kcal/mol between **5a** and **5b** is slightly larger than that between **4a** and **4b** (12.1 kcal/mol). Apparently, this is due to the more diffuse electron density on the selenium atom that weakens the resonance stabilization of the cationic center by selenium with respect to the sulfur atom.

A weaker resonance stabilization by the selenium atom can also be clearly seen by comparison of the atomic charges in the aziranium cations **4b** (vide supra) and **5b** (*q*_N_ = +0.715, *q*_CH2_ = −0.764, and *q*_CH_ = −0.220). A larger contraction of the S–CH with respect to the S–Me bond in **4b** (0.064 Å) compared to that of the Se–CH with respect to the Se–Me bond in **5b** (0.053 Å) is fully consistent with the weaker resonance of the SeMe group and explains the negative charge *q*_CH_ in **5b** and its positive value in **4b**.

**Scheme 6:**
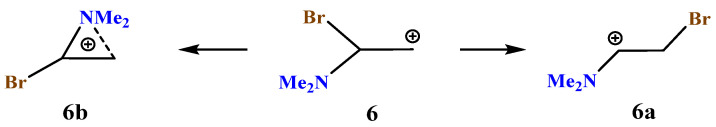
Iminium **6a** versus bromoaziranium cation **6b** formation.

**Figure 7:**
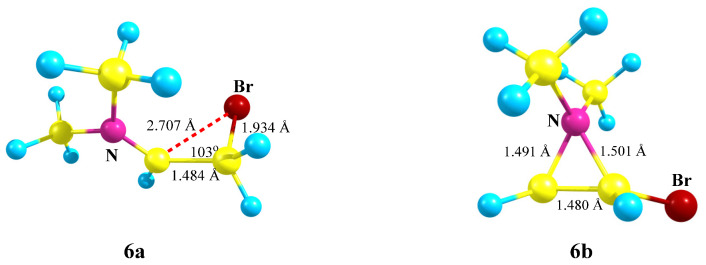
Br-assisted immonium cation **6a**, with reduced C⋯Br distance, and aziranium cation **6b**.

**X = NMe_2_, Y = Br (6).** The carbocation **6** potential energy surface profile and the structure of the global and local minima on it (Scheme 6, Figure 7) are very close to those of carbocation **5**.

The iminium ion **6a** lies lower in energy than the aziranium ion **6b** by 15.7 kcal/mol, which is very close to the energy gap for their closest analogues **5a** and **5b**. The charge on the nitrogen atom is the lowest among the above-considered aziranium cations (*q*_N_ = +0.500). The N–CH_2_ bond of 1.491 Å is slightly shorter than the N–CHBr bond (1.501 Å), in agreement with a higher electron density for the CH_2_ carbon (*q*_CH2_ = −0.692, *q*_CH_ = −0.260).

To summarize the results obtained for the amino-containing carbocations (**1**)–(**6**), one can conclude that the Me_2_N group migrates spontaneously towards the carbocationic center to form the corresponding aziranium ions only in the conformers with the *p*-orbital, C–N bond and the lone pair on the nitrogen lying in one plane. In all other cases, the second heteroatom is shifted towards the carbocationic center to form the corresponding iminium ions [Me_2_N=CH–CH_2_Y]^+^ without (Y = O, SiMe_3_, PMe_2_; tetrahedral CCY angle) or with very slight (Y = SMe, SeMe, Br; CCY angle from 99 to 103°) anchimeric assistance. The local minima lie 12–21 kcal/mol above the corresponding global minima. 

**Scheme 7:**
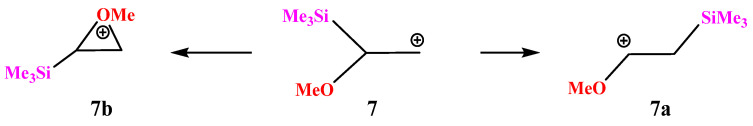
Oxenium **5a** versus (trimethysilyl)oxiranium cation **7b** formation.

**X = OMe, Y = SiMe_3_ (7).** Let us turn to combinations of oxygen with other heteroatoms. With the most electropositive heteroatom, silicon (χ = 1.74), almost full migration of the trimethylsilyl group occurs during geometry optimization, leading to cation **7a** (Scheme 7) for almost all conformations of **7** except the one with the eclipsed MeO–C bond and the *p*-orbital. In the latter case, the oxiranium ion **7b** is formed as a local minimum on the PES. 

**Figure 8:**
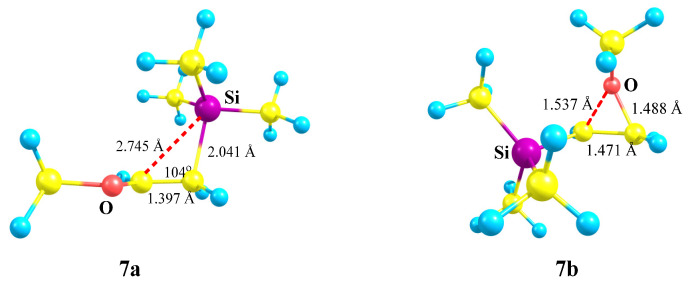
The structures of the oxenium **7a** and oxiranium **7b** cations.

The C–C–Si angle in **7a** is slightly less than the tetrahedral angle (104°, Figure 8), and the non-covalent distance C⋯Si is large (2.745 Å) but still less than the sum of the vdW radii (3.8 Å). The C–C bond length in **7a** of 1.397 Å is in between those of the double and ordinary bonds, but 0.04 Å shorter than in the nitrogen analogue **2a** (1.439 Å, Figure 3), indicating a weaker conjugation of the OMe versus the NMe_2_ group. In the oxiranium ion **7b**, the C–C distance is much longer and is close to that for the ordinary bond, indicating strong anchimeric assistance by oxygen. However, ion **7b** corresponds to a local minimum on the PES lying as much as 35.2 kcal/mol above the global minimum **7a** in energy. This is the maximum difference between the two minima on the PESs of all the studied structures. The much larger value of Δ*E* for the **7a**/**7b** pair than for the **2a**/**2b** pair could be due to the lower stability of the oxiranium **7b** relative to the aziranium ion **2b**. Geometrically, it is represented by a slightly shorter C–C bond in **7b** as compared with that in **2b** (1.471 versus 1.495 Å) and a larger sum for the two C–O bonds in **7b** as compared with the two C–N bonds in **2b** (3.025 versus 2.988 Å, Figure 3 and Figure 8). However, these effects, which are themselves moderate, should be further reduced by a stronger conjugation in the iminium **2a** than in the oxenium ion **7a**. A more probable reason for a large difference (ΔΔ*E* = 14.4 kcal/mol) between the global and local minima is that iminium ions **2a** and **7a** have principally different electronic structures. While in the aziranium ion **2b** the positive charge is localized on the Me_2_N group (mostly on nitrogen), in the oxiranium ion **7b** it is concentrated on the carbon fragment CHCH_2_ (summed with hydrogens), the OMe group bearing negative charge (−0.063 on OMe, −0.409 on oxygen).

**Scheme 8:**
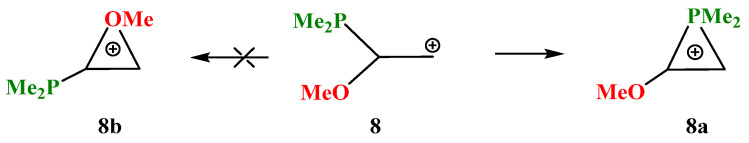
Formation of phosphiranium cation **8a** and no spontaneous optimization to the oxiranium cation **8b**.

**X = OMe, Y = PMe_2_ (8)**. The transition from silicon to its closest neighbor, phosphorus, changes the situation dramatically. Even in the most favorable migration conformation for the OMe group in carbocation **8**, only the dimethylphosphino group is shifted towards the cationic center and stops at the formation of the anchimerically stabilized phosphiranium cation **8a** (Scheme 8).

**Figure 9:**
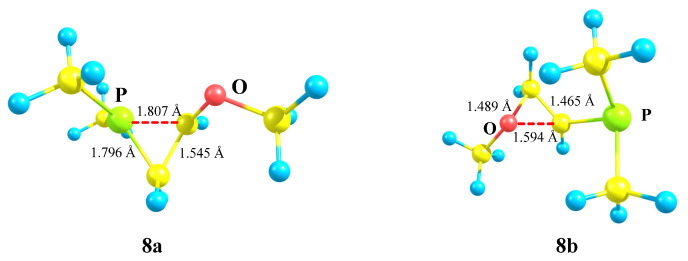
P-stabilized **8a** and O-stabilized **8b** anchimerically assisted cations.

The isomeric oxiranium cation **8b** was also located on the PES, but only starting with the “preorganized COC triangle”. Note that, unlike all the above-considered structures, no minima on the PES corresponding to open linear cations could be found. The phosphiranium **8a** and oxiranium **8b** cations look similar, but there is one principal geometrical difference: the C–C bond lengths in the almost isosceles triangle in **8a** coincide with that of normal ordinary C–C bonds (1.545 Å), whereas in the COC ring in **8b** (1.465 Å) the lengths are intermediate between those of the C–C and C=C bonds (Figure 9). The charge density distribution is also radically different. The charge on phosphorus *q*_P_ in **8a** is equal to +1.041, which allows it to be considered as a true phosphiranium ion, whereas in **8b** it is −0.399, the largest positive charge of +0.929 (summed with hydrogens) being located on the ring carbon bonded anchimerically with oxygen (*q*_C_ = 0.287). Therefore, electronically, cation **8b** is similar to **7b**, except for the fact that the charges on the ring carbons in the latter are reduced due to the electron-donating effect of the trimethylsilyl group. For the same reasons as discussed for **7b**, the oxiranium cation **8b** lies high above (28.4 kcal/mol) the phosphiranum cation **8a**.

**Scheme 9:**
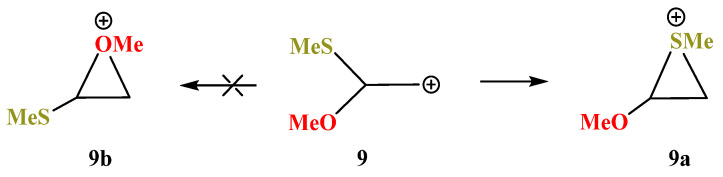
Formation of thiiranium cation **9a** and no spontaneous optimization to the oxiranium cation **9b**.

**Figure 10:**
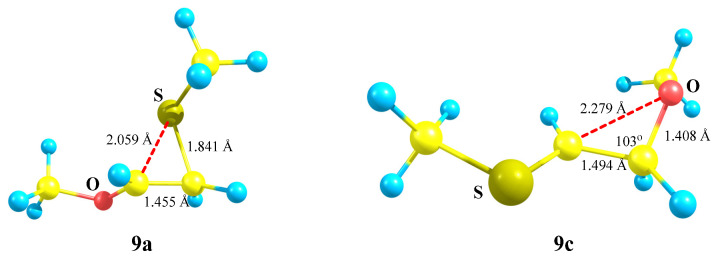
Strong anchimeric assistance in the thiiranium cation **9a** and its absence in the linear cation **9c**.

**X = OMe, Y = SMe (9).** No oxiranium cation **9b** could be localized on the PES of cation **9**. Even in the conformation with the eclipsed MeO–C bond and *p*-orbital, the MeS group shifts towards the cationic center to form the anchimerically stabilized cation **9a** (Scheme 9), which is the global minimum. Moreover, the “preorganized” oxiranium structure **9b** suffers the COC ring opening during optimization, resulting in practically linear S-stabilized cation **9c** (Figure 10), which lies 17.0 kcal/mol above the global minimum **9a**. 

Thiiranium cation **9a** has a slightly asymmetrical ring, suggesting a substantial interaction with the sulfur atom, whereas in **9c** the interaction of the cationic center with oxygen is insignificant. It follows from a large non-bonded C⋯O distance of 2.279 Å and the C–C–O angle in **9c** being only slightly different from the tetrahedral bond angle (Figure 10). 

Noteworthy is the difference in the atomic charges: in the thiiranium cation **9a**, the C–C bond is strongly polarized in the direction from the OCH to the CH_2_S group, the value of Δ*q* = *q*_CH_ − *q*_CH2_ being 0.716, and there is a zero charge on the sulfur atom. In the less stable isomeric cation **9c**, the direction of the C–C bond polarization is the same but the degree of polarization is much smaller (Δ*q* = 0.298), and the sulfur atom is positively charged (*q*_S_ = 0.085). The charge on oxygen is the same (*q*_O_ = −0.573).

**Scheme 10:**
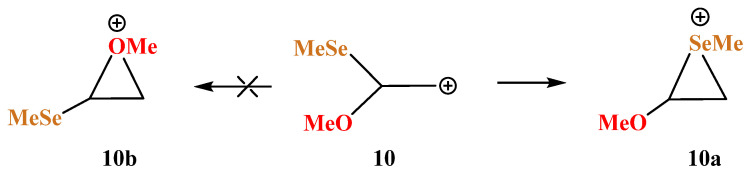
Formation of seleniranium cation **10a** and no spontaneous optimization to the oxiranium cation **10b**.

**X = OMe, Y = SeMe (10).** Selenium, the heavier analogue of sulfur, wins even more in competition with oxygen, Scheme 10; the seleniranium cation **10a** is 22.1 kcal mol more stable than the isomeric **10c**. As in the case of **9**, neither conformation of cation **10** can be optimized to the oxiranium cation **10b**, which, being taken as the starting point, is optimized to the linear cation **10c** with the C–C–Se angle even closer to the tetrahedral than in its analogue **9c**.

**Figure 11:**
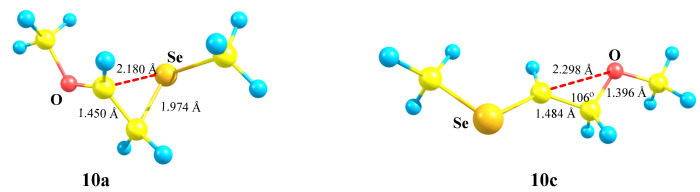
Strong anchimeric assistance in seleniranium cation **10a** and its absence in cation **10c**.

Cation **10a** in Figure 11 is geometrically very similar to **9a** in Figure 10. However, a stronger anchimeric stabilization in **10a** becomes evident from the analysis of atomic charges in the two structures. While the electron donation from the sulfur atom estimated as Δ*q*(**9c**–**9a**) is as low as 0.084, the same effect calculated as Δ*q*(**10b**–**10a**) is about three times as large as that, being 0.238, in spite of a slightly larger non-bonded C⋯Se distance and a C–C–Se bond angle. Apparently, this is also due to a more diffuse electron density on the selenium atom, as in the case of cations **5** (vide supra). 

The C–C bond in both **10a** and **10c** is polarized in the same direction as in cation **9**, but the values of Δ*q* are substantially larger: 0.850 in **10a** and 0.658 in **10c**. The electron density on selenium is much lower than in the corresponding sulfur analogues: *q*_Se_ = 0.192 in **10a** versus *q*_S_ = 0 in **9a** and *q*_Se_ = 0.430 in **10c** versus *q*_S_ = 0.298 in **9c**.

**Scheme 11:**
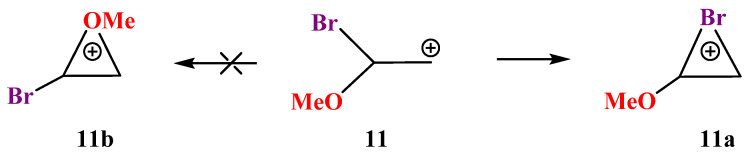
Formation of the (methoxy)bromiranium cation **11a** and no spontaneous optimization to the bromooxiranium cation **11b**.

**Figure 12:**
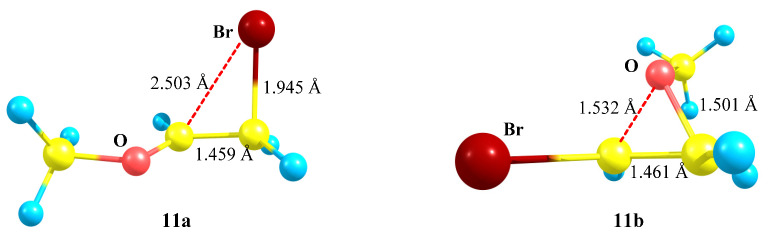
Anchimeric stabilization in the bromiranium **11a** and oxiranium **11b** cations.

**X = OMe, Y = Br (11).** Cation **11** behaves similarly to its analogues **9** and **10**. The trend of distortion of the most stable Y-stabilized iranium cation and the increase in the energy gap between the global (Y-stabilized) and local (O-stabilized) cations is maintained in going to the heaviest member of the O-containing cations in the series Y = S, Se, Br (Scheme 11, Figure 12).

Cation **11a** in Figure 12 is more asymmetric than all its analogues in the series of O-containing cations, except for cation **7a** in Figure 8, which was, apparently, due to the presence of the most electropositive SiMe_3_ group in **7a**. 

**Figure 13:**
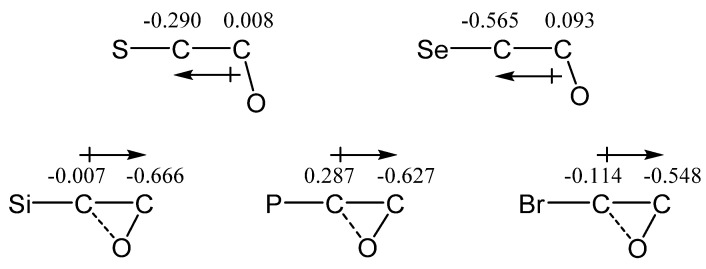
C–C bond polarization in the O,Y carbocations (Y = Si, P, S, Se, Br).

Therefore, similar to the amino-containing carbocations (**1**)–(**6**), no spontaneous migration of the MeO group to the carbocationic center occurs in carbocations (**7**)–(**11**). However, a partial or complete shift of the MeO group gives rise to the formation of local minima corresponding to the O-anchimerically assisted cations lying 17.0–35.2 kcal/mol above the Y-anchimerically assisted global minima. Moreover, the oxiranium cations are well-known species whose involvement, e.g., in the deoxyfluorination reaction of fluorocarbohydrates, has been shown and reinforced by DFT calculations [39,40]. A remarkable feature of the oxygen-containing cations (**7b**)–(**11b**) is that they can be divided into two types, depending on the C–C–O angle, and have drastically different electronic distributions in the C–C–O fragment as well as different energy gaps between the **a** and **c** isomers. The former group includes O,S and O,Se cations **9c** and **10c**, which have C–C–O angles close to tetrahedral angles (103 and 106°) and the lowest values for Δ*E* (17.0 and 22.1 kcal/mol). The C–C bonds in **9c** and **10c** are polarized in the direction from oxygen, as shown in Figure 13. The second group includes O,Si, O,P, and O,Br cations **7b**, **8b**, and **11b**, which have C–C–O angles of 63, 65, and 60°, respectively, and larger Δ*E* values equal to 35.2, 28.4, and 24.3 kcal/mol, respectively. The C–C bonds in them are polarized towards the carbon bearing the covalently bound oxygen atom, Figure 13.

The oppositely directed polarization in the two types of cations can be explained by substantial binding of the oxygen atom with the second heteroatom-bearing carbon in cations **7b**, **8b**, and **11b**. 

**Scheme 12:**
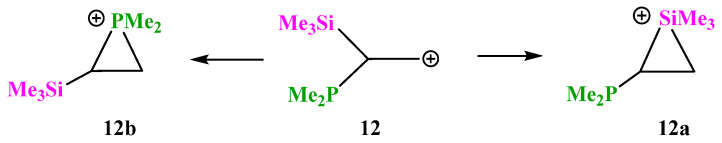
Competitive formation of siliranium **12a** and phosphiranium **12b** cations.

**X = SiMe_3_, Y = PMe_2_ (12).** Cation **12** contains in one molecule a silicon and phosphorus atoms, which are the least electronegative among the studied analogues, both being more electropositive than carbon (χ_Si_ = 1.8, χ_P_ = 2.1, and χ_C_ = 2.5). In the case of **12**, as in a number of the above-considered cations containing a group capable of the formation of iranium structures, the P-anchimerically stabilized cation **12b** is formed only if the starting conformation has the Me_2_P–C and *p*-orbital eclipsed. Otherwise, the geometry optimization results in the migration of the more electropositive (and hence less capable of anchimeric assistance) Me_3_Si group, Scheme 12.

**Figure 14:**
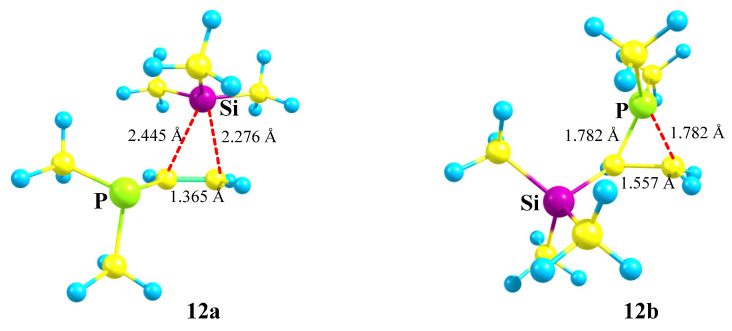
A π-complex of the Me_3_Si cation with the C=C bond **12a** and symmetric phosphiranium cation **12b**.

However, the phosphiranium cation **12b** is the global minimum on the PES lying 20.8 kcal/mol below the local minimum of the siliranium cation **12a**, and thus phosphorus wins in the competition with silicon. The C–P and C–C bond lengths in the isosceles CPC triangle in **12b** are practically equal to those of ordinary C–P and C–C bonds, proving that it is a true phosphiranium ion (Figure 14). As for cation **12a**, judging from the strongly elongated C–Si bonds and the C–C bond length close to that in alkenes, it is rather a π-complex of the Me_3_Si cation with dimethyl(vinyl)phosphine. This is independently proved by a notably larger charge on the Me_3_Si group in **12a** (*q*_Me3Si_ = +0.531) than in **12b** (*q*_Me3Si_ = +0.281).

**Scheme 13:**
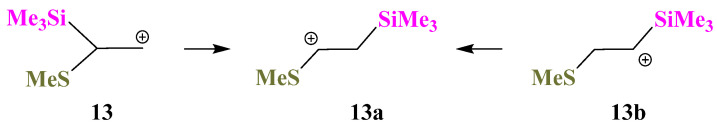
Formation of the same cation **13a** from the primary cation **13** and the secondary cation **13b**.

**X = SiMe_3_, Y = Sme (13).** Interesting results were obtained for the Si,S-containing cations **13**. The same secondary 1-(methylthio)-2-(trimethylsilyl)ethanium cation **13a** is formed by geometry optimization of either the primary 2-(methylthio)-2-(trimethylsilyl)ethanium cation **13** or the secondary 2-(methylthio)-1-(trimethylsilyl)ethanium cation **13b**, Scheme 13, and **13a** is the global minimum on the PES. The transformation **13** → **13a** occurs by migration of the Me_3_Si group to the cationic center, whereas the **13b** → **13a** conversion is the result of the sigmatropic rearrangement with 1,2-hydride shift.

**Figure 15:**
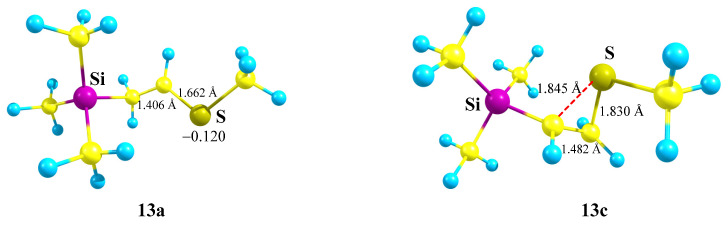
1-Methylthio-2-(trimethylsilyl)ethanium cation **13a** (global minimum) and thiiranium cation **13c** (local minimum).

The S-anchimerically stabilized cation **13c** is a local minimum on the PES formed only from the most favorable conformation of **13** with the MeS and *p*-orbital lying in one plane. Notably, the anchimerically stabilized cation **13c** is 2.82 kcal/mol less stable than the linear cation **13a**. The structures of both minima are shown in Figure 15.

The Si–C–C angle in **13a** (105°) is close to the tetrahedral angle; the S–C–C angle (124°) is close to trigonal. The structure of cation **13a** is close to perpendicular; the Si–C–C–S dihedral angle is 96°. The CCS triangle in **13c** is close to isosceles, the lengths of the two C–S bonds evidencing strong anchimeric stabilization by sulfur, which is also proved by the positive charge *q*_S_ = +0.144 in **13c**, in contrast to the negative value of *q*_S_ = −0.120 in **13a**. 

**Scheme 14:**
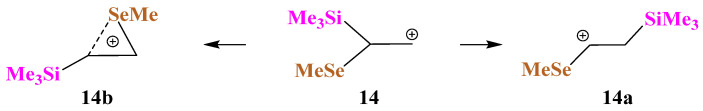
Formation of linear cation **14a** and anchimerically assisted seleniranium cation **14b**.

**X = SiMe_3_, Y = SeMe (14).** The situation changes when the sulfur atom in cation **13** is replaced by its heavier analogue, selenium, in cation **14**. As in the case of Si,S-containing cations, no Si-stabilized cation is formed, even in the form of a π-complex, but the Me_3_Si group migrates to the cationic center to form linear cation **14a**, Scheme 14.

**Figure 16:**
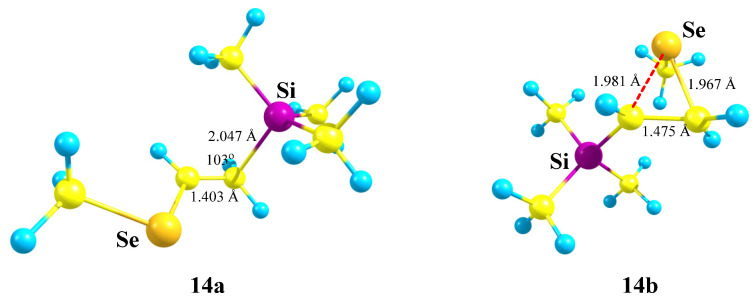
Linear (**14a**, local minimum) and Se-anchimerically stabilized (**14b**, global minimum) Si,Se cations.

As in the case of Si,S-containing cation **13c**, the selenium anchimerically stabilized cation **14b** was located on the PES by optimization of the conformation of **14** most appropriate for the shift of the MeSe group to the cationic center. Remarkably, the seleniranium cation **14b** corresponds to the global minimum, although it lies only 0.9 kcal/mol lower in energy than **14a**. The structures of the isomeric cations **14a** and **14b** are shown in Figure 16. Note the much shorter C–C bond in **14a** compared to that in **14b**, indicating a weaker stabilization of the former cationic species, which, probably, is responsible for the energetic preference of the latter. 

The inverse energy order of the linear and anchimerically assisted **14** cations with respect to the corresponding Si,S-containing **13** analogues is in agreement with the stronger anchimeric assistance of the selenium atom as compared to that of the sulfur atom.

**Scheme 15:**
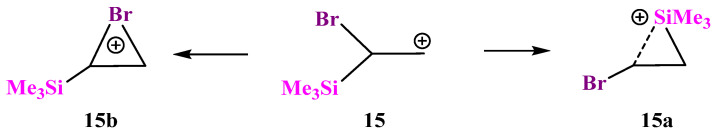
Competitive formation of π-complex **15a** of the trimethylsilyl group with vinyl bromide and the bromiranium cation **15b**.

**X = SiMe_3_, Y = Br (15).** Similar to the examples above, cation **15** can be stabilized by either the Si atom in the form of a π-complex of the trimethylsilyl cation with the double bond in vinyl bromide or by the Br atom in the form of a bromiranium cation formed from the conformation most appropriate for the bromine shift to the cationic center, Scheme 15.

**Figure 17:**
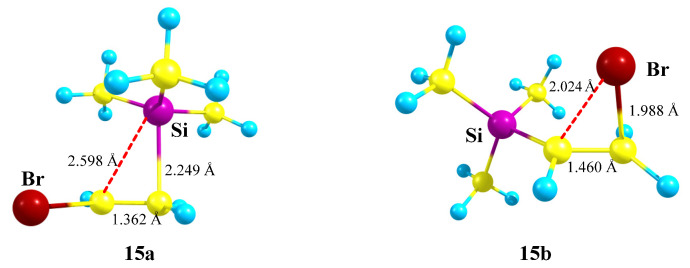
Siliranium **15a** (global minimum) and bromiranium **15b** (local minimum) cations.

The structure of π-complex **15a** (Figure 17) is very similar to that of the Si,P-containing cation **12a** (Figure 14). A drastic difference between the two is that π-complex **15a** is 12.2 kcal/mol more stable than the bromiranium cation **15b**, whereas π-complex **12a** is much less stable than the isomeric phosphiranium ion **12b** (vide supra). 

The most reasonable explanation for this difference is that stabilization of the phosphiranium ion **12b** is much stronger than that of the bromiranium ion **15b**, as evidenced by a much longer C–C bond in **12b** (1.557 Å) as compared with **15b** (1.460 Å). This, in turn, is due to the well-known tendency of phosphorus to form tetracoordinated species, along with the much less pronounced ability of bromine to expand its coordination number to 2, although bromiranium (bromonium) ions have been known for many years [41,42,43,44] and have been the subject of experimental studies [45].

Note that the higher stability of cation **15a** cannot be assigned to anchimeric assistance by the silicon atom since it has long C⋯Si bonds of 2.25–2.60 Å, a short C–C bond of 1.362 Å, and, as its analogue **12a**, is in fact a π-complex of the Me_3_Si cation with vinyl bromide. However, stabilization via the formation of a siliranium cation was proposed in the literature [46]. Thus, its formation via the migration of the Bu^t^Ph_2_Si group in N-tosylazetidines, leading to the corresponding pyrrolidines, was reported [47]. Notably, the silyl group formation was shown to be non-concerted with the C–N bond cleavage, which is in accordance with our conclusion regarding a π-complex rather than a real Si-anchimerically assisted structure of the “siliranium” ion (vide supra) [47].

**Scheme 16:**
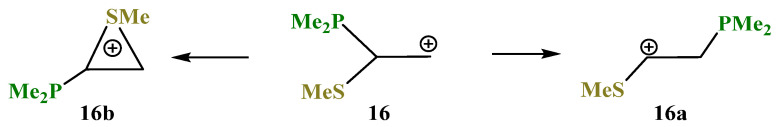
Competitive formation of linear cation **16a** versus thiiranium cation **16b**.

**Figure 18:**
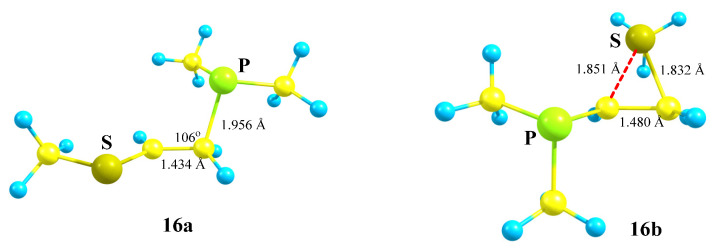
1-Methylthio-2-(dimethylphosphino)ethan-1-ium **16a** and thiiranium **16b** cations.

**X = PMe_2_, Y = SMe (16).** The phosphorus atom can either fully migrate to the adjacent carbocationic center (N,P cation **3a**, Scheme 3) or form anchimerically stabilized phosphiranium ions (O,P and Si,P cations **8a** and **12b**, Scheme 8 and Scheme 12). The optimization of the conformation of cation **16** with the eclipsed Me_2_P–C bond and *p*-orbital results in full migration of the Me_2_P group and formation of the open cation **16a**, Scheme 16, Figure 18. The same procedure for the conformation with the eclipsed MeS–C bond and *p*-orbital gives rise to the thiiranium cation **16b** lying 2.3 kcal/mol lower in energy than **16a**, and so the MeS group wins over Me_2_P in anchimerical assistance.

Note the opposite charges on the phosphorus and sulfur atoms in **16a** and **16b**: in the former, the phosphorus atom is charged positively (*q*_P_ = 0.089) and the sulfur atom negatively (*q*_S_ = −0.040), whereas in **16b** the ratio is vice versa (*q*_P_ = −0.336, *q*_S_ = 0.002). The lower electron densities on phosphorous in **16a** and on sulfur in **16b** are, respectively, due to hyperconjugation with the PMe_2_ group and the anchimeric assistance by the MeS group.

**Scheme 17:**
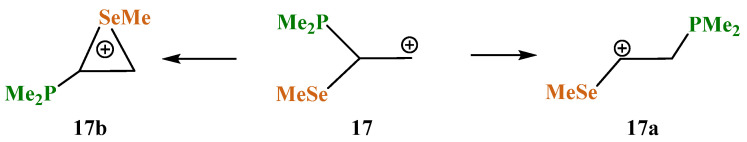
Competitive formation of linear cation **17a** versus seleniranium cation **17b**.

**Figure 19:**
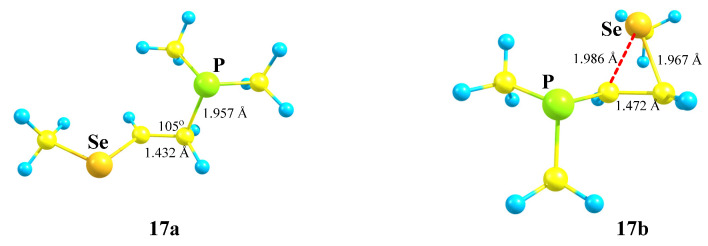
1-Methylseleno-2-(dimethylphosphino)ethan-1-ium **17a** and seleniranium **17b** cations.

**X = PMe_2_, Y = SeMe (17).** The structures and relative energies of the isomeric cations (Scheme 17, Figure 19) change only slightly in going to the heavier chalcogen, selenium, except that the energy difference between the anchimerically assisted seleniranium ion **17b** and the open 1-methylseleno-2-(dimethylphosphino)ethan-1-ium cation **17a** increases to 8.2 kcal/mol, indicating a greater energy gain in the case of selenium as compared to sulfur.

As in the P,S cations **16a** and **16b**, the phosphorus atom is charged positively in **17a** (*q*_P_ = 0.107) and negatively in **17b** (*q*_P_ = −0.378). The selenium atom is charged positively in both cations, but the electron density on it in **17b** is lower (Δ*q*_Se_ = 0.316 − 0.242 = 0.074), due to the anchimeric assistance being stronger than that of the MeS group.

**Scheme 18:**
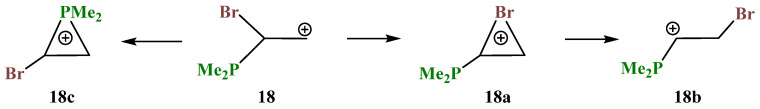
Global minimum of phosphiranium cation **18c** and successive formation of bromiranium **18a** and linear **18b** cations.

**X = PMe_2_, Y = Br (18).** Unlike cations **16** and **17**, in which the PMe_2_ group fully migrates to the cationic center and in which no phosphiranium ion could be located on the PES, the geometry optimization of cation **18** in the conformation with the eclipsed Me_2_P–C bond and the *p*-orbital gives rise to phosphiranium ion **18c** as the global minimum. Unlike all other cases, two more local minima, bromiranium ion **18a** and linear ion **18b**, were found on the PES, Scheme 18. 

The phosphiranium ion **18c** is energetically most favorable; the next favorable is the open cation **18b**, lying 8.2 kcal/mol above **18c**; and the least stable is bromiranium ion **18a**, lying high above **18c**, by 28.9 kcal/mol. 

**Figure 20:**
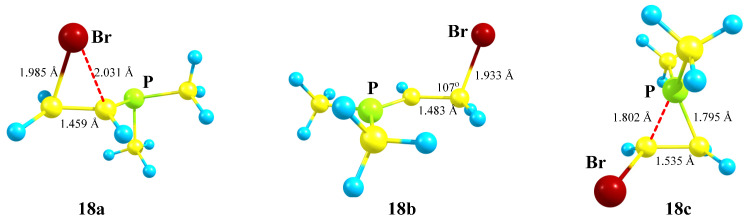
High-lying intermediate cation **18a**, linear cation with full migration of bromine **18b**, and phosphiranium ion **18c** (global minimum).

The phosphiranium ion **18c** is practically symmetrical (Figure 20), and the length of the C–C bond in it falls in the range typical for ordinary bonds. The positive charge in **18c** is located on the phosphorus atom (*q*_P_ = +1.026), and its structure is very similar to the O,P- and Si,P-containing cations **8a** and **12b** shown in Figure 9 and Figure 14. The values for *q*_P_ in cation **18b** are even larger (*q*_P_ = +1.075), which may seem strange, but this is explained by the much shorter P–CH bond (1.647 Å) and a negative charge on the adjacent CH carbon (*q*_C_ = −0.628), indicating its dipolar structure: P^+^–C^−^. The P–CH bonds in **18a** and **18c** are significantly longer (1.853 and 1.795 and 1.802 Å, respectively). The charges on bromine *q*_Br_ in **18b** and **18c** are slightly negative (−0.04), whereas in **18a** the charge is expectedly positive (+0.144). 

1-Methyl-1-phenylphosphonium triflate salt was synthesized at the end of the last century and its X-ray crystal structure was determined [48]. It contains a weakly coordinated triflate counterion and hence an almost “free” phosphiranium cation, which is structurally close to those determined in the present work. Thus, the phosphiranium ring in it is isosceles and the length of the C–P bonds is 1.76 Å (cf. Figure 9, Figure 14 and Figure 20).

**Scheme 19:**
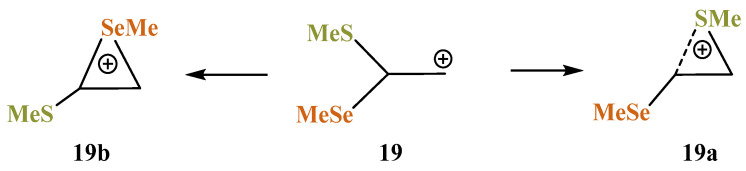
Competition between the formation of thiiranium **19a** and seleniranium **19b** cations.

**X = SMe, Y = SeMe (19).** Of particular interest is the rivalry between the sulfur and selenium atoms in cation **19**. The intermediacy of thiiranium and seleniranium ions has been confirmed by extensive studies of the chalcogenofunctionalization reactions of alkenes by Denmark et al. [49,50,51,52]. One can assume that selenium, as a more polarizable atom capable of stronger anchimeric assistance [7,13,35,53], would preferably migrate to the cationic center to form the corresponding seleniranium cation. However, the migration of a particular group X is determined by the orientation of the C–X bond with respect to the empty *p*-orbital on the carbocation center: either SMe or SeMe migrates, provided that it is eclipsed with the *p*-orbital (Scheme 19).

**Figure 21:**
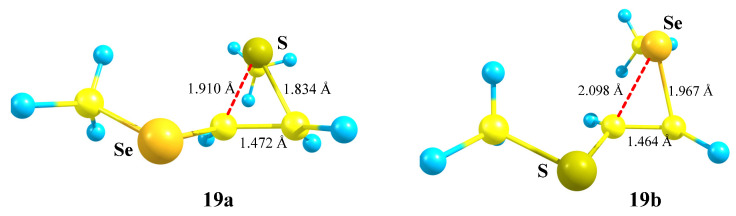
Thiiranium **19a** and seleniranium cation **19b**.

The isomeric seleniranum cation **19b** expectedly turned out to be the global minimum, lying 5.2 kcal/mol below **19a**. The structures of the two anchimerically assisted chalcogeniranium cations are presented in Figure 21.

The chalcogen atoms in the onium rings are expectedly positively charged (*q*_S_ = +0.167 in **19a**, *q*_Se_ = +0.328 in **19b**) but bear the opposite charge, not being involved in anchimeric assistance (*q*_S_ = −0.284 in **19b**, *q*_Se_ = +0.054 in **19a**).

**Scheme 20:**
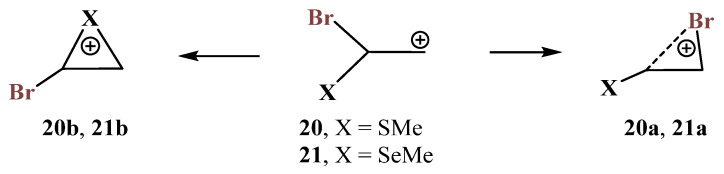
Competitive formation of bromiranium **20a** and **21a** versus chalcogeniranium **20b** and **21b** cations.

**X = SMe, Y = Br (20) and X = SeMe, Y = Br (21).** The formation and transformations of haliranium ions were summarized in an excellent review covering the literature published up to 2014 [54]. Later, the use of haliranium ions as efficient halogenating agents via halogen olefin-to-olefin transfer was reported [55,56]. For Y = Br, in the presence of most electronegative second heteroatoms N and O, the geometry optimization results in the migration of the bromine atom (Scheme 6 and Scheme 11, Figure 7 and Figure 12) to give the most stable cations. The migration of bromine was also observed in the P,Br-containing cation **18** (Scheme 18), although the formed open cation **18a** was only a local minimum. For the most electropositive silicon atom, the Me_3_Si group migrated during optimization, the bromiranium ion **15b** (Figure 16) being also a local minimum on the PES. Therefore, the combination of bromine with sulfur and selenium, as the elements with intermediate electronegativity and different polarizability, is of particular interest. In the conformations of cations **20** and **21** with the X–C and *p*-orbital eclipsed, the corresponding chalcogeniranium ions **20b** and **21b** are formed. In all other cases, the shift of bromine was observed with the formation of bromiranium ions **20a** and **21a** (Scheme 20).

**Figure 22:**
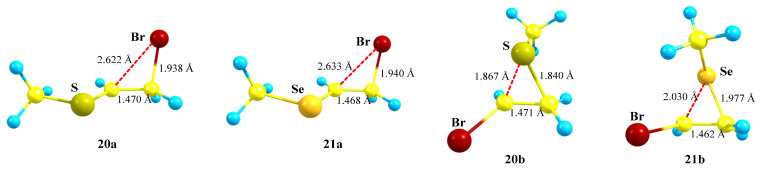
The structures of bromiranium **20a** and **21a** and chalcogeniranium **20b** and **21b** cations.

The chalcogeniranium ions **20b** and **21b** are the global minima, the isomeric bromiranium ions **20a** and **21a** being less stable by 1.1 and 8.2 kcal/mol, respectively. Expectedly, the energy difference is notably larger in the case of selenium, clearly demonstrating its higher anchimeric stabilization. Both bromiranium ions are highly asymmetric and have very similar structures, Figure 22. Interestingly, the positive charge on sulfur in the thiiranium ion **20b** (*q*_S_ = +0.222) is larger than that in the isomeric bromiranium ion **20a** (*q*_S_ = +0.114), whereas that on selenium in the seleniranium ion **21b** (*q*_Se_ = +0.388) is smaller than that in **21a** (*q*_Se_ = +0.498).

In order to give a compact overview, the results are summarized below in Table 1.

Note that, among most electropositive substituents Y in the X-stabilized three-membered ring, Y = PMe_2_ and especially Y = SiMe_3_ attenuate the demands of anchimeric assistance and decrease the total positive charge on X (*q*_X_). It is incorrect to compare the charges on the substituents with different electronegativities, but for those with practically the same electronegativity, SMe and SeMe, the values of *q*_X_ for X = Se, which are larger by ~0.1–0.2 (depending on Y), provide a semi-quantitative estimation of their anchimeric assistance abilities.

## 3. Conclusions

The routes of rearrangement and the structures and relative energies of the inherently unstable primary carbocations with two different heteroatoms in the molecule CHXY–CH_2_^+^ (X, Y = Me_2_N, MeO, Me_3_Si, Me_2_P, MeS, MeS, Br) were analyzed theoretically at the MP2/aug-cc-pVTZ level. Depending on the ability to stabilize an adjacent carbocationic center either by direct conjugation or via anchimeric assistance by a heteroatom and the relative orientation of the C–X and C–Y bonds with respect to the empty *p*-orbital on the cationic center, the complete migration or a partial shift of either X or Y or a sigmatropic 1,2-hydride shift may take place. The Me_2_N group is shifted to the cationic center to give aziranium ions only in the conformers with the *p*-orbital, C–N bond and the nitrogen lone pair lying in one plane. In other cases, the second heteroatom migrates to form the corresponding iminium ions lying 12–21 kcal/mol below the local minima. The MeO group can be partly shifted to the cationic center to give the O-anchimerically assisted cations or undergo full migration to form the oxenium cations lying 17.0–35.2 kcal/mol lower in energy. The group least capable of anchimeric assistance, Me_3_Si, either fully migrates to the cationic center, resulting in the linear secondary cations, or suffers a partial shift to form loose π-complexes of Me_3_Si^+^ with the C=C bond rather than anchimerically assisted siliranium ions, as proved by the charges on silicon and the structures of the corresponding complexes with dimethyl(vinyl)phosphine or vinyl bromide. Phosphorus in combination with chalcogens fully migrates to the cationic center to form linear cations, which, however, lie above the isomeric chalconiranium anchimerically assisted ions. Phosphorus loses to sulfur and especially selenium in the stabilization of the corresponding iranium cations. On the potential energy surface of the P,Br-containing cation, a third local minimum of the high-lying bromiranium ion can be located. For the S,Se-containing cation, as anticipated, the seleniranium cation is the global minimum on the PES. The S,Br and Se,Br cations, depending on the starting conformation, give upon optimization either the corresponding chalcogeniranium ions, as global minima, or bromiranium ions, lying, respectively, 1.1 and 8.2 kcal/mol higher in energy.

## Figures and Tables

**Table 1:** MP2/aug-cc-pVTZ optimized geometries (°, Å) and total atomic charges on X (*q*_X_) for the X-anchimerically assisted cations.

X in 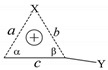	Y	α	β	*a*	*b*	*c*	*q*_X_
NMe_2_	OMe	60	60	1.496	1.490	1.489	0.759
	SiMe_3_	61	59	1.478	1.510	1.495	0.500
	PMe_2_	61	59	1.479	1.505	1.491	0.517
	SMe	62	59	1.483	1.536	1.484	0.744
	SeMe	62	59	1.483	1.525	1.484	0.792
	Br	61	60	1.491	1.501	1.480	0.744
OMe	SiMe_3_	63	59	1.488	1.537	1.471	−0.063
	PMe_2_	65	58	1.489	1.594	1.465	0.027
	Br	62	60	1.501	1.532	1.461	0.256
SiMe_3_	PMe_2_	80	67	2.276	2.445	1.365	0.531
	Br	88	60	2.249	2.598	1.362	0.816
PMe_2_	OMe	65	64	1.796	1.807	1.545	0.748
	SiMe_3_	64	64	1.782	1.782	1.557	0.460
	Br	65	65	1.795	1.802	1.535	0.838
SMe	OMe	76	60	1.841	2.059	1.455	0.209
	SiMe_3_	67	66	1.828	1.844	1.482	0.043
	PMe_2_	67	66	1.832	1.851	1.480	0.152
	SeMe	70	64	1.834	1.910	1.472	0.356
	Br	68	66	1.840	1.867	1.471	0.460
SeMe	OMe	77	62	1.974	2.180	1.450	0.303
	SiMe_3_	69	68	1.967	1.981	1.475	0.248
	PMe_2_	69	67	1.967	1.986	1.472	0.340
	SMe	74	64	1.967	2.098	1.464	0.434
	Br	71	67	1.977	2.030	1.462	0.566
Br	SiMe_3_	70	67	1.988	2.024	1.460	0.120
	PMe_2_	70	67	1.985	2.031	1.459	0.144
	SMe	100	47	1.938	2.622	1.470	0.033
	SeMe	100	46	1.940	2.633	1.468	0.023

## Supplementary Materials

The following supporting information can be downloaded at: https://www.mdpi.com/article/10.3390/molecules28010038/s1, Structures, Cartesian coordinates and energies of all calculated species.
