# Supplementary material for: Conjugative Stabilization versus Anchimeric Assistance in Carbocations
Source: Molecules. 2022 Dec 21;28(1):38. doi: 10.3390/molecules28010038 (PMC9822469; doi:10.3390/molecules28010038)
Supplement: Supplementary file 1 [file molecules-28-00038-s001.zip › molecules-2060607-supplementary.pdf]

Supporting material

Conjugative Stabilization versus Anchimeric Assistance in Carbocations

Bagrat A. Shainyan

A. E. Favorsky Irkutsk Institute of Chemistry, Siberian Division of the Russian Academy of Sciences, 1 Favorsky Street, 664033 Irkutsk, Russian Federation

Structures, MP2/cc-pVTZ optimized energies (a.u.) and Cartesian coordinates for all computed structures. The structures were confirmed to be minima through vibrational analysis.

1a

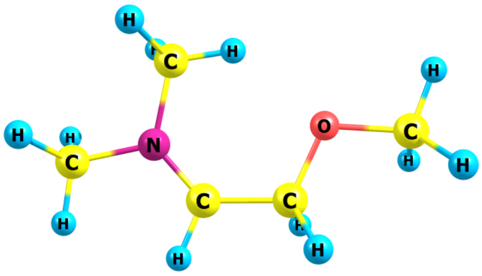

E(MP2) = –326.8187254

6	-0.492390	-0.949190	-0.000171
6	0.991483	-0.923570	-0.000429
8	1.560401	0.342968	-0.000172
7	-1.355412	0.003769	-0.000022
6	2.993765	0.262304	0.000500
6	-2.796107	-0.306934	0.000461
6	-1.067604	1.449866	-0.000304
1	-0.911598	-1.950947	-0.000062
1	1.294334	-1.518133	0.877401
1	1.294047	-1.517686	-0.878670
1	3.342458	-0.257014	0.894152
1	3.358136	1.283148	0.000417
1	3.343299	-0.257404	-0.892602
1	-3.237652	0.137272	0.889255
1	-2.944859	-1.381557	-0.000163
1	-3.238505	0.138410	-0.887332
1	-1.534976	1.871410	0.886773
1	-1.534292	1.870867	-0.888006
1	-0.000596	1.616651	0.000022

1b

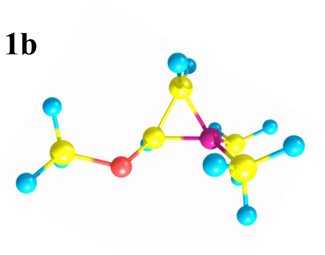

E(MP2) = –326.7732618

6	0	0.410390	-0.520402	-0.171463
6	0	-0.264144	-0.708645	1.143099
7	0	-0.961871	0.019486	0.038661
6	0	-1.137047	1.483778	0.160848
6	0	-2.077056	-0.677085	-0.633189
8	0	1.348452	0.445997	-0.323899
6	0	2.685374	-0.079246	-0.160327
1	0	0.433465	-1.351156	-0.872805
1	0	-0.710457	-1.657696	1.401868
1	0	0.044997	-0.060847	1.950933
1	0	-1.251255	1.894842	-0.838632
1	0	-0.262605	1.910852	0.637296
1	0	-2.030660	1.659062	0.754416
1	0	-2.978477	-0.526970	-0.043842
1	0	-1.849423	-1.737348	-0.699721
1	0	-2.195395	-0.255025	-1.627933
1	0	3.350621	0.760649	-0.319404
1	0	2.877082	-0.854288	-0.901707
1	0	2.812497	-0.476849	0.846286

1c

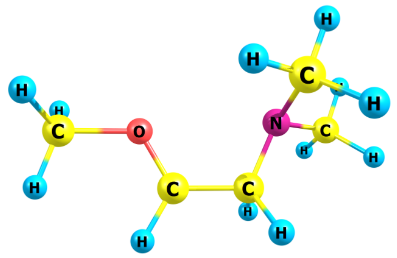

E(MP2) = –326.7732618

6	-0.996039	-0.739797	0.415339
6	0.462730	-0.883206	0.471793
7	1.190078	0.097391	-0.267985
8	-1.531606	0.170613	-0.254187
6	2.529384	-0.393709	-0.600612
6	-3.002285	0.283440	-0.330483
6	1.275630	1.359309	0.467537
1	-1.635246	-1.442100	0.956039
1	0.711853	-0.967534	1.552278
1	0.642647	-1.898907	0.083519

1	3.136056	-0.584391	0.293755
1	3.031095	0.355948	-1.207187
1	2.451373	-1.309743	-1.183432
1	-3.454984	-0.508874	0.258267
1	-3.238329	0.202313	-1.384846
1	-3.234613	1.269358	0.054148
1	1.789694	2.091543	-0.149813
1	1.825946	1.244863	1.411034
1	0.280289	1.744662	0.684182

2a

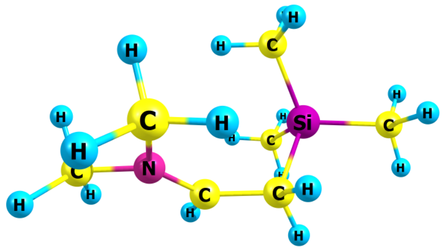

E(MP2) = –620.4688501

6	1.085888	-0.574015	-0.787423
6	-0.097481	0.079444	-1.281429
14	-1.494472	-0.016410	0.105881
7	2.113326	-0.031943	-0.205676
6	-1.744206	-1.824987	0.507458
6	-2.990306	0.781314	-0.668054
6	-0.830755	0.934716	1.576483
6	3.204010	-0.853191	0.331201
6	2.290891	1.408520	-0.020544
1	1.125197	-1.658011	-0.836897
1	0.019641	1.128155	-1.542100
1	-0.509652	-0.482063	-2.119768
1	-2.569816	-1.934564	1.212570
1	-0.862131	-2.268394	0.971682
1	-1.995053	-2.402720	-0.382975
1	-3.818310	0.778976	0.042624
1	-3.312334	0.239480	-1.557449
1	-2.792273	1.816359	-0.946887
1	-1.569455	0.928803	2.379922
1	-0.627131	1.977005	1.328218
1	0.079160	0.482015	1.974984
1	3.295709	-0.660016	1.398246
1	4.130208	-0.574803	-0.166958
1	2.989771	-1.903346	0.159987
1	2.285623	1.624638	1.046280
1	1.498045	1.960540	-0.508844
1	3.253876	1.690465	-0.441389

2b

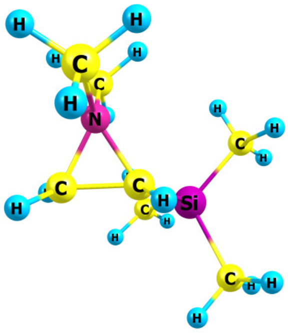

E(MP2) = –620.4357172

6	-0.444636	-0.787020	0.114147
6	-1.314640	-0.998663	-1.083394
14	1.311540	0.016200	0.086877
6	2.413820	-1.440901	0.470250
6	1.366149	1.328752	1.418751
6	1.660178	0.699923	-1.621571
7	-1.742424	-0.047977	-0.035689
6	-1.733812	1.391986	-0.350976
6	-2.862749	-0.457867	0.833129
1	-0.565831	-1.553830	0.874146
1	-1.035090	-0.524734	-2.014306
1	-1.934798	-1.880654	-1.158601
1	3.458451	-1.126655	0.462017
1	2.301160	-2.234473	-0.269104
1	2.199869	-1.855130	1.455919
1	2.395998	1.664430	1.552342
1	1.023054	0.938959	2.377898
1	0.770672	2.205897	1.166295
1	2.713026	0.987036	-1.663423
1	1.083671	1.588577	-1.877877
1	1.504496	-0.053166	-2.395307
1	-2.661418	1.635757	-0.864590
1	-0.885063	1.611225	-0.990118
1	-1.658098	1.948845	0.579643
1	-3.796983	-0.181385	0.349186
1	-2.763388	0.054373	1.787242
1	-2.820182	-1.533297	0.980165

3a

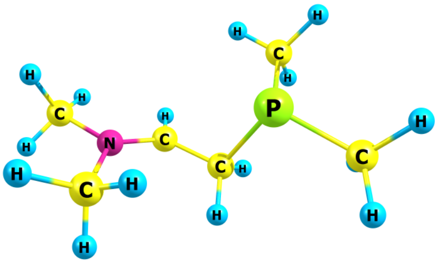

E(MP2) = –632.4474904

6	-0.952320	-0.411011	0.752700
6	0.279056	0.333169	0.989107
15	1.408973	0.115106	-0.539637
7	-1.962523	0.031376	0.078204
6	1.854711	-1.654984	-0.301210
6	2.897763	0.918713	0.175113
6	-3.158878	-0.788016	-0.147488
6	-1.986944	1.390414	-0.485169
1	-1.041879	-1.431275	1.110697
1	0.109533	1.404074	1.095360
1	0.792318	-0.057666	1.866728
1	2.670455	-1.885974	-0.986842
1	1.018328	-2.302962	-0.562171
1	2.183611	-1.863103	0.717668
1	3.721553	0.773889	-0.524365
1	3.170287	0.491700	1.140276
1	2.729010	1.989679	0.278262
1	-4.020858	-0.254542	0.247371
1	-3.047187	-1.743016	0.356119
1	-3.280619	-0.935695	-1.218162
1	-2.752684	1.422868	-1.253325
1	-1.018865	1.616583	-0.923430
1	-2.230263	2.099510	0.304624

3b

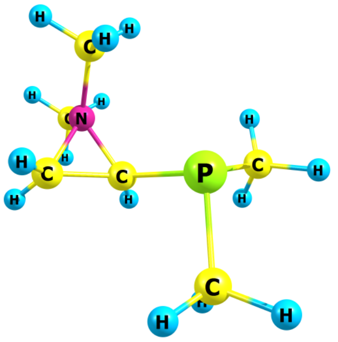

E(MP2) = –632.421868

6	0.143341	-0.406178	0.445571
6	1.050578	-1.448078	-0.117152
15	-1.334987	0.163436	-0.512139
7	1.529874	-0.051634	-0.018249
6	-2.455514	-1.150245	0.126665
6	1.730559	0.713470	-1.267079
6	2.525976	0.246784	1.030054
6	-1.819631	1.573699	0.565053
1	0.091782	-0.365991	1.530277

1	0.879638	-1.777679	-1.133223
1	1.552438	-2.140467	0.543510
1	-3.466376	-0.907328	-0.200549
1	-2.442999	-1.213578	1.214783
1	-2.185894	-2.112780	-0.305339
1	1.660396	1.773657	-1.034402
1	0.963250	0.439274	-1.983743
1	2.720205	0.472900	-1.648939
1	2.443677	1.298690	1.292765
1	3.516553	0.029671	0.636738
1	2.323650	-0.373546	1.898732
1	-2.798368	1.926744	0.240261
1	-1.115773	2.396685	0.444928
1	-1.878356	1.286926	1.615364

4a

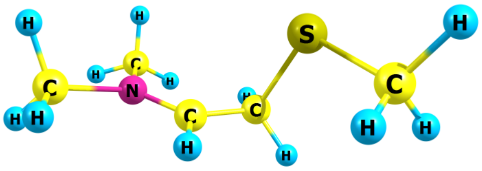

E(MP2) = –649.4341745

6	0.457675	-0.350185	0.607807
6	-0.577468	0.661521	0.864330
7	1.638379	-0.140098	0.124772
16	-1.660625	0.417655	-0.594449
6	2.498667	-1.260376	-0.277953
6	2.178014	1.190095	-0.179633
6	-2.823846	-0.792995	0.082845
1	0.190506	-1.390448	0.757341
1	-0.230295	1.688655	0.871255
1	-1.137529	0.448669	1.772265
1	2.695264	-1.174632	-1.345174
1	1.998572	-2.199779	-0.065971
1	3.434100	-1.196339	0.272508
1	1.991451	1.408371	-1.230538
1	3.248635	1.166217	0.002265
1	1.721981	1.944848	0.449041
1	-3.363480	-0.360646	0.921544
1	-2.324741	-1.714362	0.372237
1	-3.521366	-1.010715	-0.723377

4b

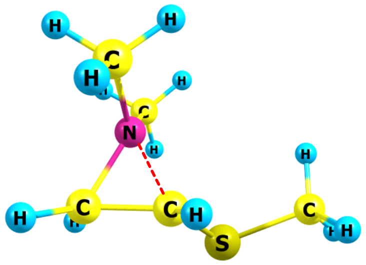

E(MP2) = –649.4148925

6	0.070356	0.467390	-0.623320
6	-1.030590	1.378122	-0.224024
16	1.599862	0.532691	0.221576
6	2.317045	-1.037311	-0.332149
1	0.091581	0.142578	-1.657704
1	-0.890475	1.980019	0.663893
1	-1.698141	1.772938	-0.976596
1	3.333101	-1.049807	0.055664
1	2.349598	-1.070328	-1.418334
1	1.768197	-1.883011	0.072474
7	-1.197295	-0.068366	0.058067
6	-1.153428	-0.512712	1.462513
6	-2.142293	-0.813044	-0.795223
1	-2.124585	-0.312886	1.909598
1	-0.370104	0.028577	1.984032
1	-0.939972	-1.578839	1.478080
1	-3.146308	-0.681235	-0.397336
1	-1.864384	-1.864056	-0.783683
1	-2.091763	-0.423125	-1.808553

5a

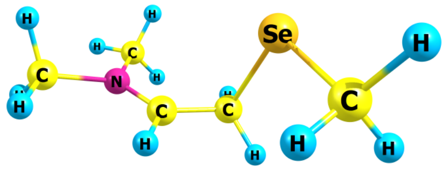

E(MP2) = –2651.8321761

6	-0.908317	0.531836	0.670031
6	0.114814	-0.382452	1.171453
7	-2.030576	0.181217	0.127333
34	1.340183	-0.366322	-0.369922
6	-2.899829	1.166977	-0.523688
6	-2.422191	-1.225601	-0.024229
6	2.430319	1.127494	0.207513
1	-0.704139	1.596300	0.694213
1	-0.208162	-1.402517	1.344543
1	0.644457	0.011116	2.034068

1	-2.945776	0.937948	-1.587191
1	-2.495223	2.163157	-0.375570
1	-3.894473	1.096319	-0.090353
1	-1.829790	-1.674072	-0.822218
1	-3.473680	-1.258748	-0.289589
1	-2.268894	-1.762628	0.906444
1	3.178797	1.257162	-0.570852
1	2.915020	0.877485	1.146343
1	1.840904	2.035391	0.289693

5b

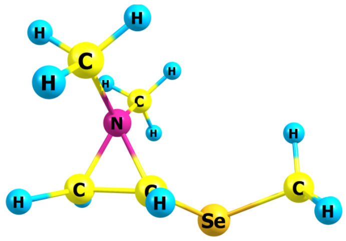

E(MP2) = –2651.8082235

6	0.344971	-0.243820	-0.734702
6	1.368001	-1.293605	-0.503305
34	-1.351069	-0.371250	0.079224
6	-1.884045	1.469625	-0.214933
7	1.606347	0.053278	0.070100
6	1.537210	0.209713	1.533840
6	2.626060	0.891418	-0.588848
1	0.385037	0.274729	-1.685359
1	1.161660	-2.050762	0.241314
1	2.042487	-1.570772	-1.300909
1	-2.924019	1.526986	0.096636
1	-1.810632	1.703125	-1.273130
1	-1.284953	2.143258	0.388949
1	2.478439	-0.135933	1.954959
1	0.703545	-0.372643	1.913757
1	1.382800	1.262466	1.758305
1	3.602695	0.627257	-0.188982
1	2.400724	1.934854	-0.382550
1	2.600969	0.706992	-1.659625

6a

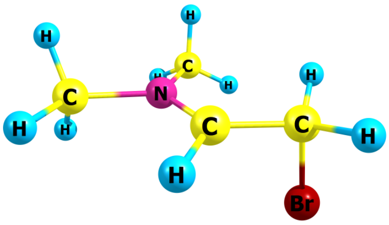

E(MP2) = –2784.5877745

6	0.810298	-0.787936	-0.502692
6	-0.406573	-0.280942	-1.168711
35	-1.646925	0.032311	0.281933
7	1.765465	-0.045063	-0.063358
6	2.934077	-0.624770	0.613547
6	1.753990	1.422449	-0.167389
1	0.906131	-1.853933	-0.328865
1	-0.266656	0.646313	-1.712660
1	-0.838182	-1.040828	-1.811617
1	3.829347	-0.266754	0.110161
1	2.883038	-1.707702	0.568182
1	2.933218	-0.284719	1.646824
1	2.298641	1.716944	-1.062494
1	2.256007	1.817792	0.710864
1	0.731826	1.784642	-0.193084

6b

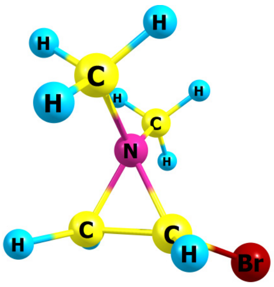

E(MP2) = –2784.5627648

6	0.144588	-0.775112	-0.117216
6	0.965230	-0.895243	1.108450
35	-1.533069	0.043044	-0.113610
7	1.375246	0.067706	0.047213
6	1.259550	1.514792	0.326752
6	2.532945	-0.296678	-0.797741
1	0.261813	-1.516952	-0.894524
1	0.602825	-0.419264	2.008614
1	1.623096	-1.744650	1.226191
1	2.173832	1.825281	0.826474
1	0.398656	1.693242	0.961408
1	1.140892	2.036607	-0.619555
1	3.440246	0.010347	-0.282944
1	2.437934	0.220598	-1.749232
1	2.537525	-1.372252	-0.952049

7a

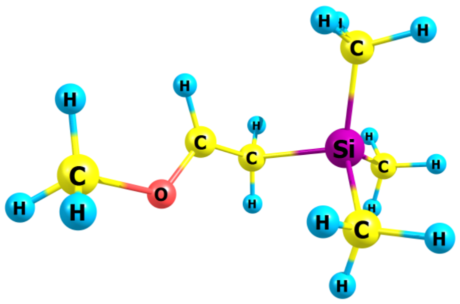

E(MP2) = –601.068947

6	1.338743	0.858454	-0.174301
6	0.147090	1.313742	0.395005
14	-1.210895	-0.149327	-0.030064
8	2.194358	0.191545	0.506245
6	-1.272625	-0.298516	-1.887700
6	-2.753041	0.565105	0.725115
6	-0.568370	-1.673682	0.825396
6	3.372289	-0.360586	-0.160438
1	1.546079	1.002665	-1.235525
1	0.134073	1.363618	1.480114
1	-0.286537	2.163586	-0.124991
1	-2.087700	-0.969734	-2.165162
1	-0.354450	-0.717348	-2.300746
1	-1.465882	0.663148	-2.364052
1	-3.576698	-0.136393	0.577858
1	-3.028960	1.508684	0.255028
1	-2.637309	0.725951	1.796556
1	-1.314341	-2.467042	0.746560
1	-0.386642	-1.493954	1.885146
1	0.350942	-2.044866	0.372257
1	3.321113	-1.436406	-0.035145
1	4.232900	0.049513	0.355113
1	3.366563	-0.080308	-1.210544

7b

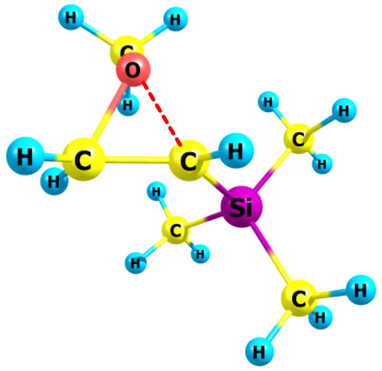

E(MP2) = –601.0129212

6	0.610446	-0.805352	-0.591865

6	1.678372	-1.383175	0.239086
14	-1.005576	0.079121	0.023510
6	-2.278190	-1.194379	-0.468818
6	-1.159553	1.682897	-0.919519
6	-0.933923	0.307070	1.879185
8	2.012404	-0.176573	-0.565991
6	2.221551	1.081529	0.168860
1	0.541313	-1.253986	-1.578665
1	1.683243	-1.177331	1.301622
1	2.255837	-2.230812	-0.100636
1	-3.268151	-0.846159	-0.169492
1	-2.105220	-2.153349	0.020332
1	-2.294209	-1.351844	-1.547338
1	-2.154444	2.099233	-0.751884
1	-1.044449	1.529698	-1.992853
1	-0.437576	2.431329	-0.594081
1	-1.935303	0.577518	2.221422
1	-0.267033	1.104054	2.207020
1	-0.662470	-0.615008	2.394906
1	3.262293	1.066345	0.474943
1	1.548686	1.123011	1.019263
1	2.024091	1.870658	-0.547346

8a

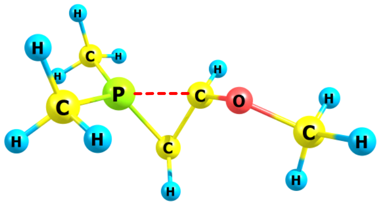

E(MP2) = –613.0469329

6	0.700761	0.585340	0.264203
6	0.180064	0.759374	-1.180334
8	1.629602	-0.402545	0.489283
15	-0.971537	-0.025515	-0.046954
6	2.960922	0.045817	0.164100
6	-2.391039	0.912208	0.525864
6	-1.146488	-1.806261	-0.156029
1	0.792977	1.498630	0.851421
1	0.622986	0.099978	-1.918098
1	-0.054292	1.759790	-1.523119
1	3.616520	-0.792315	0.370932
1	3.231282	0.896392	0.789981
1	3.024552	0.321171	-0.889247
1	-3.211683	0.790166	-0.179876
1	-2.112875	1.963053	0.586616

1	-2.687022	0.550684	1.509374
1	-1.848738	-2.062028	-0.947284
1	-1.502171	-2.184550	0.801218
1	-0.160612	-2.216763	-0.368696

8b

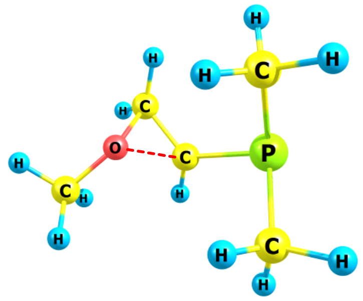

E(MP2) = –613.0017007

6	-0.418133	0.057362	-0.787323
6	-1.327688	1.170858	-0.508485
8	-1.376759	0.063825	0.486191
15	1.381129	0.088106	-0.556025
6	-2.570163	-0.798766	0.415401
6	1.603086	-1.587679	0.168292
6	1.536481	1.172003	0.918956
1	-0.799923	-0.695879	-1.470378
1	-0.945936	2.068299	-0.042407
1	-2.248796	1.271730	-1.067808
1	-2.271762	-1.744789	0.852661
1	-2.876883	-0.913072	-0.621625
1	-3.332031	-0.301825	1.007391
1	2.660505	-1.712486	0.398028
1	1.331303	-2.344620	-0.566325
1	1.023395	-1.720384	1.080934
1	2.571595	1.097235	1.252456
1	0.883586	0.868632	1.736302
1	1.360581	2.212305	0.650575

9a

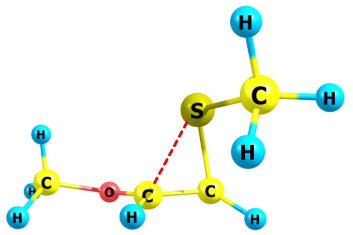

E(MP2) = –630.0370521

6	0.566052	0.353391	0.514722
6	-0.431487	1.364478	0.198966
8	1.721273	0.412706	-0.086808
16	-1.034717	-0.133640	-0.685806

6	2.555585	-0.774159	0.022340
6	-2.238269	-0.759927	0.512782
1	0.449640	-0.289917	1.384610
1	-0.120044	2.133421	-0.498224
1	-1.113393	1.677870	0.979345
1	3.535616	-0.472595	-0.324652
1	2.138718	-1.548850	-0.617442
1	2.595712	-1.097146	1.060382
1	-3.102643	-0.101329	0.508714
1	-1.794165	-0.811235	1.504365
1	-2.515437	-1.756316	0.177394

9c

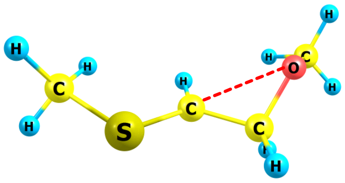

E(MP2) = –630.0099744

6	0.141532	0.045694	0.372932
6	-1.089972	-0.798322	0.317089
8	-1.914916	-0.114562	-0.596039
16	1.576770	-0.522802	-0.123369
6	-2.917657	0.692698	0.040056
6	2.722518	0.866682	-0.015420
1	0.038979	1.089037	0.658573
1	-0.878945	-1.798072	-0.062955
1	-1.532780	-0.882069	1.314954
1	-3.470015	1.171882	-0.760498
1	-2.467915	1.455708	0.679414
1	-3.582734	0.062855	0.631256
1	3.517636	0.570438	0.665226
1	2.203685	1.750373	0.342822
1	3.124568	1.020671	-1.014520

10a

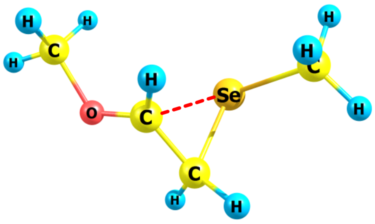

E(MP2) = –2632.4370805

6	0.925188	0.476328	0.566640
6	-0.037826	1.487617	0.175199
8	2.040946	0.385531	-0.106605

34	-0.863765	-0.175446	-0.494267
6	2.825120	-0.813576	0.135035
6	-2.012937	-0.511072	1.026723
1	0.846884	-0.022039	1.531042
1	0.255916	2.133240	-0.643620
1	-0.650075	1.937335	0.946203
1	3.788822	-0.629257	-0.322370
1	2.324228	-1.654748	-0.340199
1	2.930171	-0.971509	1.206304
1	-2.420825	-1.508623	0.886709
1	-2.803038	0.233375	1.029792
1	-1.428899	-0.472650	1.942469

10c

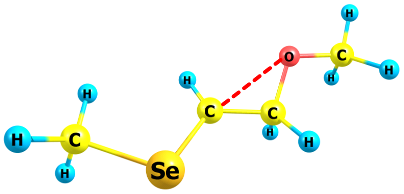

E(MP2) = –2632.4018794

6	-0.200088	0.406858	0.256194
6	-1.506989	-0.287437	0.372590
8	-2.433674	0.537298	-0.267188
34	1.297370	-0.429152	-0.071971
6	-3.769367	0.035706	-0.122430
6	2.582172	1.008964	0.002449
1	-0.199484	1.487638	0.374527
1	-1.474885	-1.301438	-0.047120
1	-1.716972	-0.384694	1.452800
1	-4.413728	0.734508	-0.643383
1	-4.044682	-0.010226	0.932616
1	-3.855574	-0.954398	-0.572695
1	3.166918	0.871297	0.908051
1	2.052335	1.956155	0.005415
1	3.210526	0.909403	-0.878524

11a

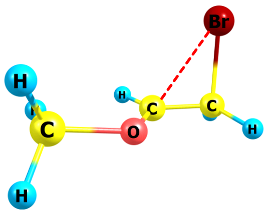

E(MP2) = –2765.1763244

6	-0.960997	0.558417	0.413097
6	0.216325	1.275858	-0.064678

35	1.349954	-0.303589	-0.014682
8	-1.866330	0.236997	-0.398151
6	-2.981150	-0.626036	0.039416
1	-1.042068	0.266798	1.460301
1	0.134337	1.642319	-1.081243
1	0.608082	1.997476	0.643962
1	-3.880782	-0.109413	-0.271776
1	-2.931175	-0.756864	1.116234
1	-2.851213	-1.560122	-0.495416

11b

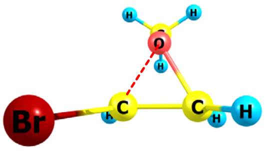

E(MP2) = –2765.1376794

6	0.333422	0.224998	0.549118
6	1.146906	1.298463	-0.016522
8	1.421801	-0.103474	-0.477419
35	-1.377342	-0.129857	-0.035655
6	2.652810	-0.751409	0.037746
1	0.593340	-0.180639	1.518198
1	0.749915	1.882315	-0.834778
1	1.957468	1.717103	0.565312
1	3.437266	-0.454459	-0.649630
1	2.451348	-1.815726	-0.004910
1	2.844412	-0.408125	1.051020

12a

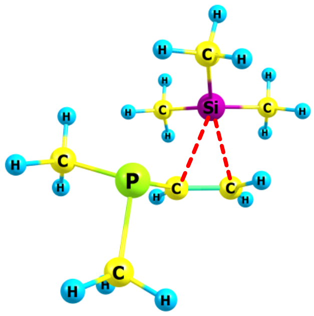

E(MP2) = –906.6596027

6	0.554719	0.647621	-0.649944
6	-0.312870	1.652923	-0.333437
14	-1.646312	-0.150416	0.054354
15	1.814386	0.019481	0.542069
6	-1.559765	-1.405903	-1.312590
6	-3.142815	0.952218	-0.051220
6	-1.255263	-0.745747	1.763040
6	2.205424	-1.588806	-0.265172

6	3.167649	1.063318	-0.168347
1	0.574002	0.299711	-1.682861
1	-0.320034	2.072023	0.669523
1	-0.886311	2.167397	-1.096208
1	-2.409409	-2.082517	-1.185859
1	-0.649302	-2.001473	-1.284034
1	-1.654481	-0.938712	-2.292804
1	-4.005525	0.326722	0.196524
1	-3.293778	1.348236	-1.054034
1	-3.107348	1.772554	0.663520
1	-2.150327	-1.257474	2.129439
1	-1.037934	0.076104	2.444170
1	-0.425866	-1.449073	1.789543
1	3.141908	-1.952166	0.158859
1	2.323519	-1.493083	-1.345005
1	1.436784	-2.326923	-0.039427
1	4.097489	0.732361	0.295473
1	3.003721	2.106057	0.096992
1	3.252982	0.960126	-1.249780

12b

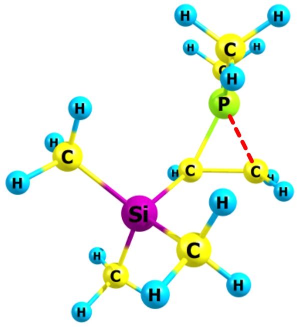

E(MP2) = –906.6927723

6	-0.130409	-0.875815	0.086269
6	-0.903881	-1.101483	-1.245951
14	1.575805	0.012709	0.128737
6	1.525974	1.235920	1.544169
6	2.829252	-1.343920	0.396734
6	1.830287	0.853318	-1.527275
1	-0.196479	-1.720256	0.770740
1	-0.498716	-0.615364	-2.125128
1	-1.354474	-2.070650	-1.422118
1	2.503968	1.701148	1.676126
1	1.267024	0.741992	2.481664
1	0.805482	2.035678	1.366698
1	3.837710	-0.927862	0.406081
1	2.782672	-2.088407	-0.398562
1	2.668323	-1.848731	1.349745

1	2.802964	1.349204	-1.520101
1	1.088336	1.618102	-1.758609
1	1.845220	0.129906	-2.343648
15	-1.702814	-0.049506	-0.050143
6	-3.113770	-0.721876	0.835273
6	-1.785699	1.722853	-0.326540
1	-3.160490	-0.282803	1.830778
1	-2.989443	-1.800359	0.915826
1	-4.028274	-0.495998	0.288602
1	-1.818634	2.234131	0.634773
1	-2.682100	1.955091	-0.900142
1	-0.902683	2.035861	-0.878958

13a

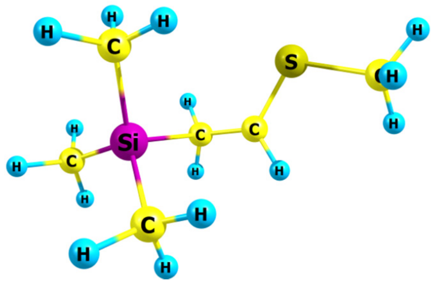

E(MP2) = –923.6739944

6	-1.021193	-0.028964	-0.912111
6	0.188858	-0.686576	-1.193493
14	1.525766	0.093749	0.135217
16	-2.215387	-0.648574	0.064554
6	1.400992	1.949774	-0.001882
6	3.127122	-0.607875	-0.503934
6	1.014190	-0.574402	1.796272
6	-3.389065	0.725966	0.197277
1	-1.185161	0.968819	-1.313699
1	0.229562	-1.752919	-0.982141
1	0.646081	-0.390764	-2.135585
1	2.205520	2.408742	0.575923
1	0.458368	2.327399	0.395601
1	1.508023	2.285856	-1.033935
1	3.939448	-0.299118	0.157212
1	3.351852	-0.241809	-1.505416
1	3.106950	-1.697226	-0.523679
1	1.743962	-0.252642	2.542548
1	0.995386	-1.664547	1.802578
1	0.039465	-0.205016	2.114278
1	-4.313243	0.292611	0.570687
1	-3.554940	1.157619	-0.785370
1	-3.011216	1.460147	0.902322

13c

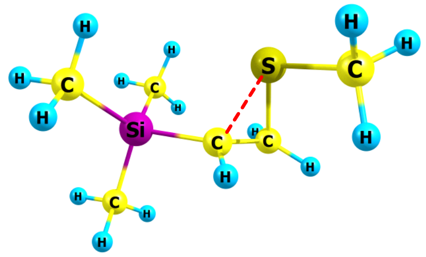

E(MP2) = –923.669494

6	0.402208	0.250153	-0.674530
14	-1.382372	-0.109053	-0.020191
16	1.747861	-0.103979	0.537457
6	-2.509293	0.759808	-1.226564
6	3.153814	-0.563797	-0.505297
6	-1.559037	-1.965790	-0.021201
6	-1.444422	0.623543	1.700647
6	1.175776	1.442601	-0.256199
1	0.683927	-0.164733	-1.639674
1	-3.548835	0.611960	-0.929326
1	-2.394016	0.365284	-2.236247
1	-2.320405	1.833259	-1.250035
1	3.174347	-1.649530	-0.555437
1	4.053400	-0.186109	-0.025333
1	3.026304	-0.132469	-1.494681
1	-2.557925	-2.245693	0.316545
1	-0.841036	-2.436454	0.651639
1	-1.421114	-2.379155	-1.020756
1	-2.415360	0.397683	2.145537
1	-1.342844	1.709441	1.695102
1	-0.686654	0.201869	2.363525
1	1.924051	1.872004	-0.911193
1	0.739309	2.113955	0.472573

14a

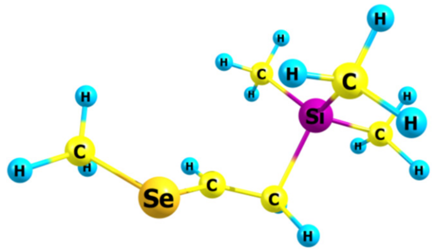

E(MP2) = –2926.0648408

6	-0.534818	0.168212	-0.978725
6	0.639759	-0.530453	-1.296077
14	1.951237	0.092627	0.146722

34	-1.872479	-0.462544	0.032740
6	1.759613	1.941275	0.305488
6	3.580942	-0.445473	-0.573832
6	1.440797	-0.845363	1.671340
6	-2.947073	1.141494	0.192313
1	-0.642461	1.194889	-1.320830
1	0.637993	-1.609013	-1.153841
1	1.140779	-0.189232	-2.199418
1	2.537647	2.329522	0.965543
1	0.796676	2.212150	0.739935
1	1.866692	2.443658	-0.656623
1	4.375421	-0.229829	0.143622
1	3.805418	0.090421	-1.495697
1	3.593298	-1.516076	-0.776602
1	2.084923	-0.549886	2.502219
1	1.549094	-1.921213	1.532795
1	0.412988	-0.630354	1.964569
1	-3.953318	0.808890	0.430525
1	-2.942830	1.661882	-0.760370
1	-2.550659	1.755759	0.993850

14b

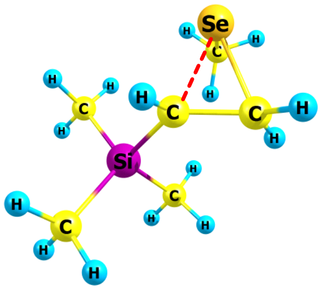

E(MP2) = –2926.0662408

6	-0.038807	0.966059	-0.170726
6	0.845389	1.361245	0.941475
14	-1.656788	-0.087112	-0.045694
34	1.773166	0.181792	-0.329479
6	-2.969426	1.144703	-0.537329
6	-1.874881	-0.631226	1.732887
6	-1.507311	-1.505007	-1.252899
6	1.598260	-1.507161	0.591910
1	-0.066246	1.705391	-0.971414
1	0.726899	0.872269	1.901177
1	1.292220	2.347393	0.961402
1	-3.953823	0.677186	-0.481716
1	-2.824201	1.494807	-1.559670
1	-2.974595	2.009535	0.126765
1	-2.878815	-1.048618	1.837348

1	-1.809123	0.215537	2.417826
1	-1.180105	-1.401726	2.065842
1	-2.462739	-2.026689	-1.328320
1	-0.760588	-2.237203	-0.946374
1	-1.249943	-1.143853	-2.249493
1	1.599261	-2.285403	-0.165727
1	0.671278	-1.509251	1.153889
1	2.458565	-1.602399	1.248534

15a

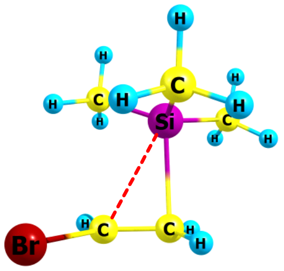

E(MP2) = –3058.8077

6	0.658232	1.055051	-0.389039
6	-0.256360	1.527991	0.502272
14	-1.576221	-0.216721	-0.017887
35	2.016850	-0.098866	0.033991
6	-1.253445	-0.838263	-1.736580
6	-3.159206	0.730603	0.180494
6	-1.122771	-1.370539	1.356029
1	0.624791	1.342574	-1.433486
1	-0.145454	1.336742	1.563216
1	-0.905794	2.334896	0.183601
1	-1.998529	-1.612988	-1.939057
1	-0.269329	-1.292725	-1.841030
1	-1.380916	-0.060293	-2.488690
1	-3.981278	0.015802	0.081996
1	-3.279486	1.485904	-0.595239
1	-3.235818	1.199369	1.160339
1	-1.751248	-2.260153	1.257749
1	-1.313709	-0.930712	2.333839
1	-0.084587	-1.693073	1.298435

15b

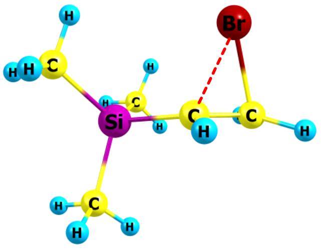

E(MP2) = –3058.7882653

6	0.136700	0.598179	-0.761624
6	1.020556	1.558575	-0.107119
14	-1.493028	-0.092627	0.040777
35	1.806252	-0.264850	-0.010050
6	-1.552135	-1.896887	-0.415989
6	-2.811448	0.949120	-0.769606
6	-1.314999	0.211271	1.874046
1	0.269240	0.544884	-1.840379
1	0.797779	1.870290	0.905488
1	1.673333	2.194770	-0.692447
1	-2.493565	-2.331190	-0.076115
1	-0.743506	-2.456885	0.054905
1	-1.493623	-2.039583	-1.495359
1	-3.788476	0.652792	-0.383364
1	-2.826817	0.809419	-1.850519
1	-2.679872	2.009895	-0.555828
1	-2.180868	-0.212950	2.385934
1	-1.291760	1.274059	2.117469
1	-0.430347	-0.270539	2.292859

16a

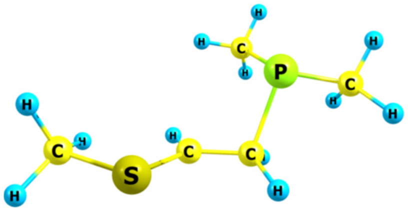

E(MP2) = –935.6474477

6	-0.852519	0.182855	0.708935
6	0.397729	0.884543	0.711747
15	1.520806	-0.038369	-0.597147
16	-2.081707	0.581826	-0.306292
6	1.600431	-1.698635	0.195177
6	3.117897	0.651880	-0.012039
6	-3.322456	-0.708711	-0.051847
1	-0.976036	-0.696815	1.335399
1	0.344477	1.907084	0.342204
1	0.910647	0.804746	1.668796
1	2.412790	-2.243122	-0.288463
1	0.683586	-2.261352	0.022580
1	1.811264	-1.641669	1.263922
1	3.905220	0.173744	-0.597706
1	3.295500	0.459345	1.045583
1	3.155143	1.722070	-0.209850
1	-4.277961	-0.207301	0.078412
1	-3.072372	-1.292491	0.829023
1	-3.343519	-1.329511	-0.943859

16b

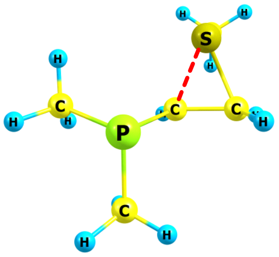

E(MP2) = –935.6511891

6	-0.126884	-0.318306	0.384081
6	-0.960401	-1.446155	-0.088470
16	-1.622389	0.189302	-0.580569
15	1.447842	0.042555	-0.531479
6	-2.864866	0.482797	0.704217
6	2.515615	-1.027916	0.521283
6	1.791752	1.695713	0.192338
1	-0.235878	-0.027131	1.427191
1	-0.617564	-2.001871	-0.953366
1	-1.612315	-1.969191	0.599889
1	-2.897490	1.555501	0.877323
1	-2.588217	-0.049054	1.610479
1	-3.817767	0.128444	0.318469
1	3.549834	-0.850267	0.225333
1	2.291507	-2.077280	0.334762
1	2.408293	-0.805065	1.582802
1	2.788858	2.000365	-0.125584
1	1.756372	1.685666	1.281827
1	1.083667	2.425933	-0.198532

17a

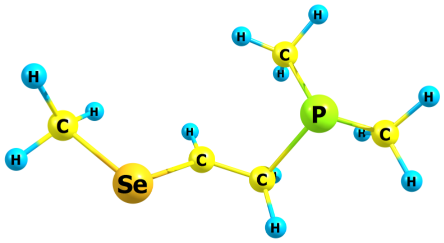

E(MP2) = –2938.0382325

6	0.358097	-0.061933	0.848652
6	-0.858305	-0.817257	0.843686
15	-1.934676	-0.004473	-0.574754
34	1.718366	-0.420663	-0.227026
6	-2.002771	1.729040	0.042460
6	-3.568372	-0.598474	0.012303
6	2.886364	1.077584	0.131945
1	0.431721	0.834420	1.458113
1	-0.758792	-1.854720	0.529918

1	-1.422390	-0.707574	1.768360
1	-2.782184	2.238450	-0.526020
1	-1.064707	2.249533	-0.147467
1	-2.255499	1.784617	1.102116
1	-4.322497	-0.167214	-0.648839
1	-3.780420	-0.295640	1.037268
1	-3.623050	-1.682420	-0.076958
1	2.928451	1.683847	-0.768147
1	3.865558	0.671116	0.367172
1	2.489420	1.641463	0.970398

17b

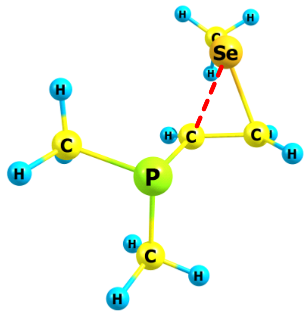

E(MP2) = –2938.0513367

6	0.284861	-0.413944	0.473085
6	-0.517100	-1.544812	-0.022204
34	-1.381273	0.173799	-0.433415
15	1.785246	0.080040	-0.495481
6	-2.574137	0.255882	1.091740
6	2.960381	-1.000892	0.424021
6	2.092778	1.698597	0.316563
1	0.214671	-0.185081	1.535123
1	-0.198547	-2.031712	-0.936596
1	-1.110736	-2.137462	0.662801
1	-2.670523	1.301932	1.367769
1	-2.143624	-0.323200	1.903501
1	-3.529902	-0.155610	0.780057
1	3.967650	-0.756020	0.086041
1	2.771432	-2.045799	0.181919
1	2.901321	-0.850193	1.501952
1	3.058624	2.070502	-0.025030
1	2.109966	1.617014	1.403514
1	1.333571	2.416889	0.008043

18a

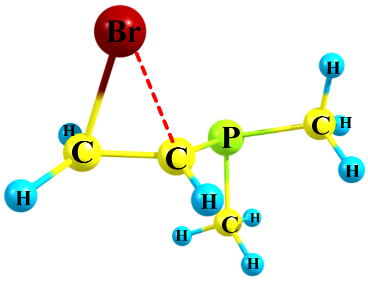

6	0	-0.121444	0.466258	0.520453
6	0	0.793000	1.506861	0.061878
35	0	1.691874	-0.260308	-0.035710
15	0	-1.491672	-0.107905	-0.586554
6	0	-2.704848	1.073919	0.154848
6	0	-1.918095	-1.646818	0.320161
1	0	-0.163528	0.312266	1.597663
1	0	1.314145	2.128513	0.779211
1	0	0.679572	1.880044	-0.949360
1	0	-3.682854	0.809156	-0.249742
1	0	-2.480281	2.092328	-0.158753
1	0	-2.747318	1.011938	1.242120
1	0	-2.854649	-2.023166	-0.091822
1	0	-2.043917	-1.480083	1.389721
1	0	-1.153380	-2.402969	0.145092

18b

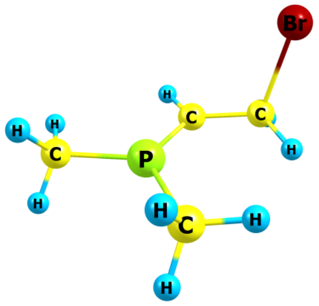

E(MP2) = –3070.8014342

6	0.438814	-0.621709	-0.703335
6	-0.743762	0.184067	-1.095067
35	-2.076279	-0.058621	0.283995
15	1.845542	-0.025852	-0.086810
6	3.159953	-1.134525	0.420253
6	2.087932	1.725827	0.207761
1	0.379573	-1.702681	-0.793145
1	-0.545693	1.247690	-1.176775
1	-1.172947	-0.183711	-2.022979
1	4.073388	-0.878257	-0.115112
1	2.862069	-2.154428	0.184037
1	3.321850	-1.030542	1.492858

1	2.949067	2.073803	-0.361640
1	2.267160	1.877961	1.272256
1	1.194559	2.267734	-0.094865

18c

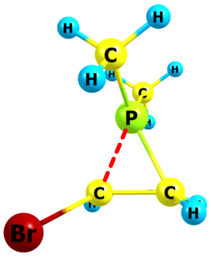

E(MP2) = –3070.8145787

6	-0.140034	-0.866804	-0.128203
6	0.569794	-1.031569	1.222797
15	1.390802	0.065242	0.062739
35	-1.780683	0.063231	-0.166505
6	1.255704	1.831579	0.327176
6	2.864903	-0.522441	-0.774932
1	-0.109519	-1.713972	-0.802382
1	0.097779	-0.557302	2.075100
1	1.034418	-1.990262	1.420638
1	0.280224	2.032819	0.767009
1	1.340172	2.339875	-0.632729
1	2.049187	2.158321	0.997656
1	3.739753	-0.283389	-0.171247
1	2.940540	-0.041056	-1.748929
1	2.787137	-1.601340	-0.899537

19a

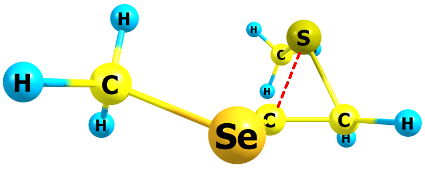

E(MP2) = –2955.0356387

6	0.288085	0.251569	0.607161
6	1.210102	1.359071	0.309308
16	1.665225	-0.129886	-0.660839
34	-1.464073	0.336414	-0.058446
6	3.009559	-0.792947	0.355473
6	-1.857837	-1.550102	0.116545
1	0.478384	-0.340247	1.496923
1	0.855162	2.156374	-0.332610
1	1.968066	1.628361	1.034366
1	3.916204	-0.245887	0.108877

1	3.111952	-1.843885	0.097305
1	2.760703	-0.679502	1.408157
1	-2.895322	-1.665343	-0.186737
1	-1.743835	-1.855235	1.152298
1	-1.215888	-2.120085	-0.548907

19b

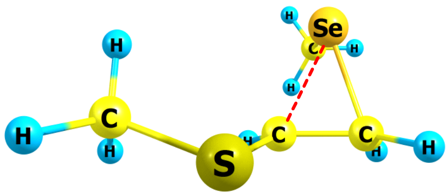

E(MP2) = –2955.0439313

6	-0.569696	0.408235	0.668654
6	0.405797	1.471678	0.423730
34	1.084230	-0.121406	-0.509100
16	-2.104841	0.480134	-0.111741
6	2.308014	-0.710128	0.870408
6	-2.662036	-1.220225	0.151481
1	-0.469743	-0.180831	1.575679
1	0.133816	2.249631	-0.279837
1	1.067643	1.768368	1.227955
1	3.175031	-0.056609	0.857164
1	2.584643	-1.732266	0.627005
1	1.800364	-0.673644	1.831206
1	-3.674109	-1.268852	-0.242914
1	-2.674670	-1.450999	1.213454
1	-2.021813	-1.906509	-0.398089

20a

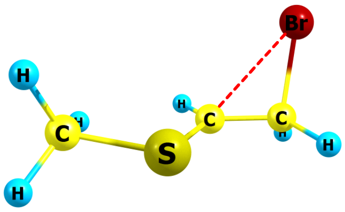

E(MP2) = –3087.7843773

6	-0.707224	0.399521	0.577128
6	0.513417	1.164062	0.281693
35	1.735599	-0.298005	-0.070172
16	-1.956722	0.352297	-0.468020
6	-3.130488	-0.808768	0.263616
1	-0.750162	-0.169628	1.501704
1	0.451106	1.795226	-0.598990
1	0.882389	1.702823	1.150087
1	-4.113920	-0.354223	0.169894

1	-2.878776	-0.983026	1.305276
1	-3.083280	-1.726644	-0.318240

20b

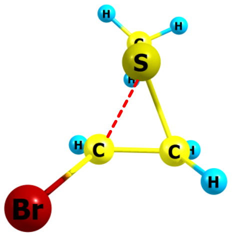

E(MP2) = –3087.7861051

6	0.094261	0.243551	0.570253
6	0.812618	1.387032	-0.012488
16	1.451490	-0.227226	-0.621693
35	-1.641648	-0.149256	0.011398
6	2.866990	-0.504474	0.472858
1	0.333824	-0.074511	1.577249
1	0.321157	1.940245	-0.803163
1	1.532972	1.924100	0.592059
1	2.995668	-1.580899	0.554811
1	2.674944	-0.058893	1.445487
1	3.732064	-0.047127	-0.002013

21a

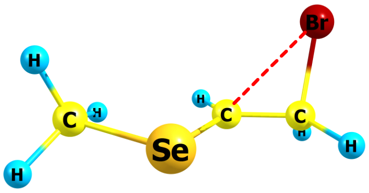

E(MP2) = –5090.1745917

6	-0.258754	0.154401	0.753818
6	0.930153	1.004148	0.611320
35	2.180610	-0.273181	-0.141511
34	-1.669345	0.326398	-0.278195
6	-2.767528	-1.141763	0.326207
1	-0.244208	-0.629458	1.505813
1	0.820287	1.834274	-0.079326
1	1.328125	1.322528	1.571282
1	-3.781007	-0.758528	0.407065
1	-2.400904	-1.485358	1.288198
1	-2.709157	-1.920384	-0.429584

21b

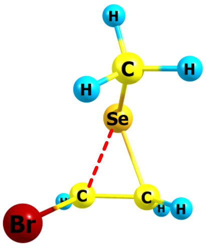

E(MP2) = –5090.1876915

6	-0.325576	0.939795	0.187422
6	0.504076	1.235632	-0.979029
34	1.518816	0.095597	0.277306
35	-1.824901	-0.150225	0.056545
6	1.161099	-1.639283	-0.481962
1	-0.380098	1.673597	0.982373
1	0.288333	0.717696	-1.905675
1	0.953394	2.218108	-1.057835
1	1.756047	-2.332055	0.108596
1	1.477696	-1.637951	-1.519924
1	0.098839	-1.848696	-0.373621
